# Transcription factors GAF and HSF act at distinct regulatory steps to modulate stress-induced gene activation

**DOI:** 10.1101/gad.284430.116

**Published:** 2016-08-01

**Authors:** Fabiana M. Duarte, Nicholas J. Fuda, Dig B. Mahat, Leighton J. Core, Michael J. Guertin, John T. Lis

**Affiliations:** 1 Department of Molecular Biology and Genetics, Cornell University, Ithaca, New York 14835, USA;; 2 Department of Biochemistry and Molecular Genetics, University of Virginia, Charlottesville, Virginia 22908, USA

**Keywords:** heat shock, transcription regulation, transcription factors, Pol II pausing

## Abstract

The coordinated regulation of gene expression at the transcriptional level is fundamental to development and homeostasis. Inducible systems are invaluable when studying transcription because the regulatory process can be triggered instantaneously, allowing the tracking of ordered mechanistic events. Here, we use precision run-on sequencing (PRO-seq) to examine the genome-wide heat shock (HS) response in *Drosophila* and the function of two key transcription factors on the immediate transcription activation or repression of all genes regulated by HS. We identify the primary HS response genes and the rate-limiting steps in the transcription cycle that GAGA-associated factor (GAF) and HS factor (HSF) regulate. We demonstrate that GAF acts upstream of promoter-proximally paused RNA polymerase II (Pol II) formation (likely at the step of chromatin opening) and that GAF-facilitated Pol II pausing is critical for HS activation. In contrast, HSF is dispensable for establishing or maintaining Pol II pausing but is critical for the release of paused Pol II into the gene body at a subset of highly activated genes. Additionally, HSF has no detectable role in the rapid HS repression of thousands of genes.

## Abstract (precis)

Here, Duarte et al. investigated the genome-wide heat shock (HS) response in *Drosophila* using precision run-on sequencing (PRO-seq) to assay the genome-wide distribution of transcriptionally engaged Pol II before and after HS in *Drosophila* S2 cells. This study comprehensively characterizes the transcriptional HS response and identifies the primary HS response genes and the rate-limiting steps in the transcription cycle that GAGA-associated factor (GAF) and HS factor (HSF) regulate.

The heat-shock (HS) response in *Drosophila melanogaster* has been an effective model system to discover and study mechanisms of transcription and its regulation (Guertin et al. 2010). This highly conserved protective mechanism (Lindquist and Craig 1988) is regulated at the transcriptional level by the HS transcription factor (HSF) (Wu 1995). When activated by stress, HSF potently activates expression of HS genes, resulting in the accumulation of molecular chaperones, the HS proteins (HSPs), which helps the cell cope with stress-induced protein aggregation and misfolding (Lindquist and Craig 1988).

The transcriptional HS response has been studied largely using *Hsp70* as a model gene (Guertin et al. 2010). *Hsp70* maintains a promoter-proximally paused RNA polymerase II (Pol II) molecule 20–40 base pairs (bp) downstream from the transcription start site (TSS) that is released to transcribe the gene at a low level during normal nonstress conditions (Rougvie and Lis 1988; Rasmussen and Lis 1993). The transcription factor GAGA-associated factor (GAF) is bound to the promoter of *Hsp70* prior to HS, and GAF is important for the establishment and stability of paused Pol II (Lee et al. 1992, 2008; Kwak et al. 2013). GAF has a key role in keeping the promoter region open and free of nucleosomes (Tsukiyama et al. 1994; Fuda et al. 2015), which allows the recruitment of general transcription factors and the initiation of transcription by Pol II. Upon HS induction, HSF trimerizes and is rapidly recruited to the promoter, where it binds to its cognate HS DNA elements (HSEs) (Xiao and Lis 1988). After binding, HSF directly and indirectly recruits coactivators and other factors (Lis et al. 2000; Saunders et al. 2003; Ardehali et al. 2009) that affect the chromatin structure and composition and promote the release of Pol II from the paused complex into productive elongation. This transition from the paused state into productive elongation depends critically on the positive elongation factor P-TEFb and has been shown to be a very general step that is essential for the regulation of virtually all genes across different species (Rahl et al. 2010; Jonkers et al. 2014). The net result of this molecular cascade is an increase in transcription levels that can be ∼200-fold for some of the HS-regulated genes (Lis et al. 1981).

Although the independent mechanisms of promoter-proximal pausing and escape to productive elongation have been well studied in the context of HS activation of *Hsp70*, we lack a comprehensive characterization of the genome-wide changes in transcription that result from HS in *Drosophila*. A thorough characterization of the affected genes is necessary to determine the generality and diversity of the roles of transcription factors such as GAF and HSF in the HS response and provide the statistical power to assess mechanisms of transcription regulation.

Previous studies have mapped HSF-binding sites during normal growth conditions and after HS and observed that HSF recruitment to a promoter is not necessary or sufficient to direct HS gene activation (Trinklein et al. 2004; Guertin and Lis 2010; Gonsalves et al. 2011). Nonetheless, the rules governing the specificity of activation and repression across the *Drosophila* genome remain incomplete. Transcriptional changes after HS have also been measured in *Drosophila* and other organisms (Leemans et al. 2000; Guhathakurta et al. 2002; Murray et al. 2004; Trinklein et al. 2004; Sørensen et al. 2005; Gonsalves et al. 2011; Vihervaara et al. 2013); however, these studies were limited in resolution both temporally and spatially by measuring steady-state levels of mature mRNA. Furthermore, measurement of mRNAs cannot distinguish the effects on mRNA stability (Lindquist and Petersen 1990) and pre-mRNA processing (Yost and Lindquist 1986; Shalgi et al. 2014) from transcription or primary from secondary effects of the HS response.

To overcome these limitations, we queried the genome-wide distribution of transcriptionally engaged RNA polymerases before and after HS induction using the precision nuclear run-on and sequencing (PRO-seq) assay and quantified differentially expressed genes. PRO-seq has high sensitivity and high spatial and temporal resolution, providing an unprecedented comprehensive view of the transcriptional profiles of cell populations. We show that the HS response is rapid and pervasive, with thousands of genes being repressed after 20 min of HS, and hundreds of genes being activated; moreover, the activated genes are not limited to the classical HSP genes. Promoter-proximal pausing is highly prevalent among the activated genes prior to HS, and here we demonstrate that its establishment on a subset of genes is dependent on GAF binding upstream and proximal to the TSS. Moreover, GAF depletion abrogates pausing and consequently impairs HS activation, indicating that this step in early transcription elongation is essential for gene activation. We also show that the recently identified transcription factor motif 1-binding protein (M1BP) (Li and Gilmour 2013) has a role in pausing and HS activation of a subset of genes that exhibit GAF-independent pausing. Furthermore, we demonstrate that only a relatively small fraction of HS-activated genes is regulated by HSF, and HS activation of these HSF-dependent genes is regulated at the level of pausing release. This study provides a genome-wide view of HS-induced transcriptional regulation and an understanding of how promoter context affects this process.

## Results

### *Drosophila* transcriptional HS response is rapid and pervasive

**Figure 1.**
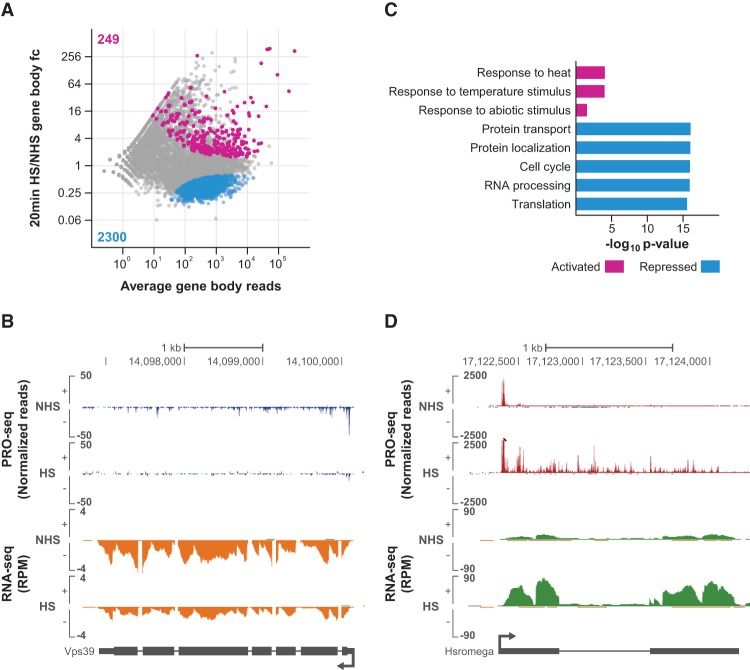
*Drosophila* transcriptional HS response is rapid and pervasive. (*A*) DESeq2 analysis of PRO-seq gene body reads between 20-min HS-treated and NHS cells displayed as an MA plot. Significantly changed genes were defined using a false discovery rate (FDR) of 0.001. Activated genes that passed our upstream transcription filter (see the Materials and Methods) are labeled in magenta, and repressed genes are labeled in blue. The number of genes in each class is shown in the plot. (fc) Fold change. (*B*) Representative view of a HS-repressed gene in the University of California at Santa Cruz (UCSC) genome browser (Kent et al. 2002). PRO-seq normalized reads for the plus strand are shown in red, and those for the minus strand are shown in blue. RNA sequencing (RNA-seq) reads for the plus strand are shown in green, and those for the minus strand are shown in orange. Gene annotations are shown at the *bottom*. (*C*) Gene ontology terms enriched in the HS-activated and HS-repressed classes. (*D*) Representative view of a HS-activated gene in the UCSC genome browser (Kent et al. 2002). Axes are the same as in *B*.

We measured nascent transcription levels by PRO-seq in *Drosophila* S2 cells prior to HS (non-HS [NHS]) and 20 min after an instantaneous and continuous HS stress (Fig. 1A; Supplemental Table S1). PRO-seq maps the active sites of transcriptionally engaged RNA polymerase complexes by affinity purification and sequencing of nascent RNAs after a terminating biotin-NTP is incorporated during a nuclear run-on experiment (Kwak et al. 2013). The density of sequencing reads is proportional to the number of transcriptionally engaged polymerase molecules present at each position when the nuclei were isolated. PRO-seq has base-pair resolution, is strand-specific, and is not affected by the background levels of accumulated RNAs (Kwak et al. 2013). Biological replicates were highly correlated for both promoter and gene body PRO-seq reads (Spearman's coefficient ranged between 0.96 and 0.99) (Supplemental Fig. S1A,B, left panels). The expected genome-wide changes in transcription that occur during HS made it unfeasible to use total number of reads to normalize our data sets between conditions. Therefore, we normalized our libraries using a set of genes previously shown to have the same Pol II ChIP-seq (chromatin immunoprecipitation [ChIP] combined with high-throughput sequencing) signals in NHS and HS *Drosophila* S2 cells (Supplemental Fig. S2), where consistent backgrounds of ChIP-seq provide a basis of normalization (see the Materials and Methods; Supplemental Fig. S3 for the normalization method and our validation tests; Teves and Henikoff 2011).

We used DESeq2 (Love et al. 2014) to identify genes whose gene body reads significantly change after HS using a false discovery rate (FDR) of 0.001 (Supplemental Table S2). Due to the compactness of the *Drosophila* genome, some of the genes identified as differentially expressed by DESeq2 appear to be false positives caused by changes in run-through transcription originating at the upstream gene. To minimize the number of false positives, we implemented a filter to exclude from our analyses genes that have high levels of transcription in the region immediately upstream of the TSS (see the Materials and Methods; Supplemental Fig. S4 for a description of the implemented filter and validation tests). The genes that passed the filter were classified as activated or repressed (Supplemental Table S2). We observed a widespread shutdown of transcription, with 2300 genes being significantly repressed after HS (Fig. 1A [blue points], B [an example of a repressed gene, *Vps39*]). This finding is in agreement with low-resolution studies in *Drosophila* salivary gland polytene chromosomes that have shown that total Pol II levels and transcription decrease in response to HS (Spradling et al. 1975; Jamrich et al. 1977). A previous Pol II ChIP-seq study in *Drosophila* S2 cells has also observed a genome-wide decrease of Pol II levels in gene bodies (Teves and Henikoff 2011). Not surprisingly, measurements of steady-state mRNA levels before and after HS, including microarray studies and our own RNA sequencing (RNA-seq) data (Supplemental Fig. S5; Supplemental Table S3), were unable to detect a genome-wide shutdown of transcription despite having the sensitivity to detect a decrease in mRNA levels for some genes (Fig. 1B). Measurements of mRNA do not detect genome-wide transcriptional repression because the reduction of mRNA levels are obscured by steady-state levels of mRNAs already present in the cells; these mRNAs have much longer half-lives than the short HS time points examined here. Overall, our results greatly expand on these previous studies, identifying and quantifying the individual genes whose transcription is repressed after HS using a base-pair resolution method that specifically maps transcriptionally engaged RNA polymerase molecules.

Gene ontology (GO) analysis reveals that the HS-repressed class is enriched for genes involved in basic cellular processes, such as cell cycle, RNA processing, protein transport and localization, and translation (Fig. 1C). This is consistent with previous findings in mammalian cells (Murray et al. 2004; Trinklein et al. 2004) and is expected as cells enter into a defensive nongrowth condition triggered by HS stress.

Although not as abundant as the repressed class, hundreds of genes are activated by HS, many very highly (Fig. 1A [magenta points], D [an example of an activated gene, *Hsromega*]). Notably, we found that all seven classical HSP genes in our gene list show strong inductions after 20 min of HS and are among the top 10 genes with the highest HS induction (Supplemental Table S2), with fold changes ranging from 44-fold to 384-fold. Consistent with this result, GO analysis reveals that the HS-activated class is enriched for genes involved in the response to temperature and abiotic stimuli (Fig. 1C). Besides the classical HSP genes, our data reveal the activation of many genes that were not previously associated with the HS response and provide a comprehensive characterization and quantification of genes whose transcription is directly activated by HS.

We measured nascent transcription levels as a function of time after HS induction to determine how fast activated and repressed genes respond to HS (Supplemental Fig. S6; Supplemental Table S1). Biological replicates produced high correlations for PRO-seq reads within either the promoter or gene body regions (Spearman's coefficient ranged between 0.9 and 0.99) (Supplemental Fig. S1C,D). The sequential HS time points displayed a progressive increase in the number of genes that were significantly activated (Supplemental Fig. S6A [magenta points], B [an example of an activated gene, *CG13321*]) and repressed (Supplemental Fig. S6A [blue points], C [an example of a repressed gene, *CG14005*]) by HS. No genes are significantly different after 30 sec, and only a small number of genes are significantly different after 2 min of HS. We observed a substantial genome-wide response to HS as early as 5 min after HS; the response is even more pervasive at later time points. Previous studies have shown that classical HSP genes are activated very rapidly (O'Brien and Lis 1993), but here we demonstrate that many other genes have a rapid response for both activation and repression. The number of significantly activated and repressed genes further increases after 10 and 20 min of HS (Supplemental Fig. S6A). Overall, our results demonstrate that the HS response produces an immediate and primary change in the transcription levels of ∼27% (∼24% repressed and ∼3% activated) of the unambiguously mappable mRNA-encoding genes (Core et al. 2012), with the repression of thousands of genes and the activation of hundreds of genes.

### Activated genes are highly paused prior to HS

**Figure 2.**
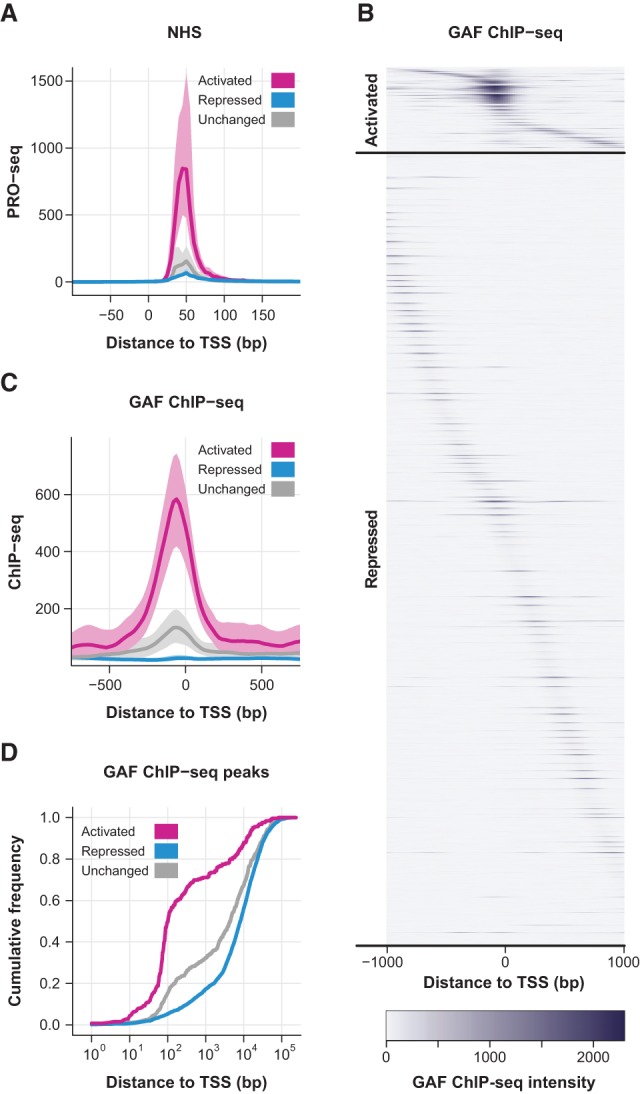
GAGA factor is highly enriched in the promoter region of HS-activated genes prior to HS. (*A*) PRO-seq read density between −100 and +200 bp to the TSS (in 5-bp bins) for the LacZ-RNAi NHS data set of HS-activated (*n* = 249), HS-repressed (*n* = 2300), and unchanged (*n* = 517) genes. The shaded area represents the 75% confidence interval. (*B*) Heat map showing the GAF ChIP-seq signal in 20-bp windows from ±1 kb to the TSS of HS-activated (*n* = 249) and HS-repressed (*n* = 2300) genes. For each class, genes were ordered by the distance between the highest-intensity window and the TSS. (*C*) GAF ChIP-seq read density between −750 and +750 bp to the TSS (in 20-bp bins) of HS-activated (*n* = 249), HS-repressed (*n* = 2300), and unchanged (*n* = 517) genes. The shaded area represents the 75% confidence interval. (*D*) Cumulative distribution plots of the distance between the closest GAF ChIP-seq peak and the TSS of each gene in the HS-activated, HS-repressed, and unchanged classes.

During normal cell growth, classical HSP genes have a paused, transcriptionally engaged polymerase between 20 and 50 bp downstream from the TSS (Rougvie and Lis 1988; Rasmussen and Lis 1993). Furthermore, promoter-proximal Pol II pausing is the major regulatory step for the HS activation of the *Hsp70* gene, where it maintains the promoter region open and accessible to transcription factors (Lee et al. 1992; Shopland et al. 1995). We used our PRO-seq data to determine whether promoter-proximal pausing is a common feature of HS-activated genes. The average PRO-seq read intensity profile across HS-activated genes reveals a strong peak in the promoter-proximal region, which is substantially higher than repressed or unchanged gene classes (Fig. 2A). DNase I hypersensitivity data (Kharchenko et al. 2011) indicate that the promoter region of HS-activated genes is more accessible than the other two classes under basal uninduced conditions (Supplemental Fig. S7A). These data are consistent with the notion that promoter-proximal pausing is important to maintain an open chromatin environment around the TSS.

We calculated the pausing index (PI)—which is the ratio of read density in the promoter-proximal region relative to the gene body—for each individual gene (Supplemental Table S4; Core et al. 2008). The vast majority of HS-activated genes (∼90%) was classified as paused (Fisher's exact, *P*-value ≤ 0.01) (Supplemental Fig. S7B; Core et al. 2008). The PI is significantly higher for activated genes compared with the repressed (Mann-Whitney *U*-test, *P*-value < 2.2 × 10^−16^) and unchanged (Mann-Whitney *U*-test, *P*-value < 2.2 × 10^−16^) classes (Supplemental Fig. S7C). Although the pausing levels of HS-repressed genes are not as high as the activated class (Fig. 2A), a considerable percentage of repressed genes were also classified as paused (∼80%) (Supplemental Fig. S7B). Overall, our results indicate that high levels of promoter-proximal pausing are a general feature of HS-induced genes prior to HS and may play an important role in poising these genes for HS activation by transcription factors.

### GAGA factor is highly enriched in the promoter region of HS-activated genes

To identify candidate factors that play a role in allowing genes to be HS-activated, we screened modENCODE and other publically available genomic transcription factor-binding data (Celniker et al. 2009; Li and Gilmour 2013; Fuda et al. 2015) for factors that are differentially enriched in HS-activated relative to HS-repressed or unchanged genes prior to HS (Supplemental Table S5). The most significant differential enrichment was observed for GAF (Fig. 2B; Supplemental Table S5; GAF ChIP-seq data from Fuda et al. 2015). As seen in Figure 2B, when compared with the repressed class, HS-activated genes show enriched GAF binding immediately upstream of the TSS, which is also evidenced by a peak in the average ChIP-seq intensity profile (Fig. 2C). Furthermore, de novo motif analysis identified the DNA sequence bound by GAF, the GAGA element (Omichinski et al. 1997; Wilkins 1998), as the most significantly overrepresented motif in the promoter region of HS-activated genes (Supplemental Fig. S8).

We then identified the closest GAF ChIP-seq peak to the TSS of each gene and plotted the cumulative distribution of these distances for our three gene classes (activated, repressed, and unchanged) (Fig. 2D). GAF binds significantly closer to the activated genes than the repressed (Kolmogorov-Smirnov test, *P*-value < 2.2 × 10^−16^) (Fig. 2D) and unchanged (Kolmogorov-Smirnov test, *P*-value < 2.2 × 10^−16^) (Fig. 2D) classes, and >70% of activated genes are bound by GAF within ±1 kb of the TSS. These results suggest that GAF binding close to the TSS prior to HS is important for the activation of HS-induced genes.

### GAF is critical for HS activation when bound immediately upstream of the core promoter

**Figure 3.**
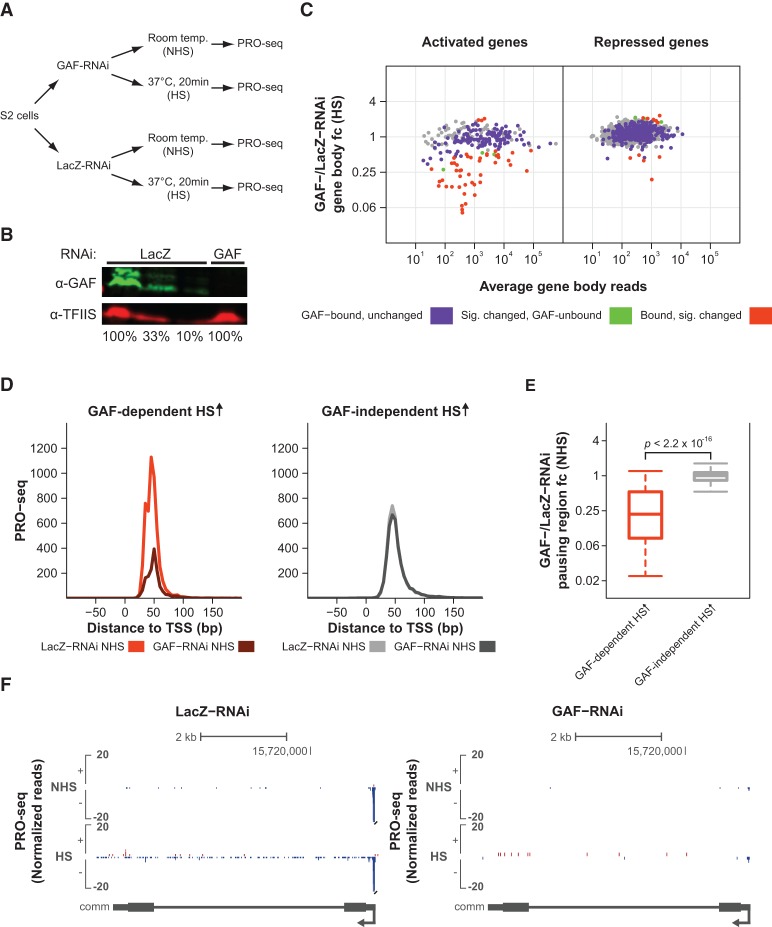
GAF's role in HS activation correlates with its function in establishing promoter-proximal pausing prior to HS. (*A*) Experimental setup. *Drosophila* S2 cells were treated with either GAF-RNAi or LacZ-RNAi for 5 d. Nuclei were then isolated for PRO-seq after cells were incubated at room temperature (NHS) or heat-shocked for 20 min (HS). (*B*) Western blot of whole-cell extracts from LacZ-RNAi-treated and GAF-RNAi-treated cells using antibodies detecting GAF and TFIIS (loading control). One-hundred percent is equivalent to 1.5 × 10^6^ cells. (*C*) DESeq2 analysis to determine the effect of GAF-RNAi treatment on the PRO-seq gene body reads after HS for the HS-activated (*n* = 249) and HS-repressed (*n* = 2300) classes. DESeq2 was used to identify significantly changed genes between GAF-RNAi HS and LacZ-RNAi HS cells, and the results are displayed as MA plots. Significantly changed genes were defined using an FDR of 0.001. GAF-bound genes are labeled in purple, significantly changed genes (according to DESeq2) are labeled in green, and genes that are both GAF-bound and significantly changed are labeled in orange. (fc) Fold change. (*D*) PRO-seq read density between −100 and +200 bp to the TSS (in 5-bp bins) for the LacZ-RNAi NHS and GAF-RNAi NHS treatments of genes with GAF-dependent (*n* = 44) or GAF-independent (*n* = 199) HS activation (HS ↑). (*E*) Box plot showing the GAF-/LacZ-RNAi pausing region fold change prior to HS (NHS) for genes with GAF-dependent or GAF-independent HS activation. Mann-Whitney *U*-test, *P*-value < 2.2 × 10^−16^. Over 70% of the genes with GAF-dependent HS activation have significantly reduced pausing upon GAF depletion prior to HS, while only 15% of the GAF-independent genes were significantly affected. (*F*) Representative view in the UCSC genome browser (Kent et al. 2002) of a gene with GAF-dependent pausing prior to HS whose activation is inhibited by GAF-RNAi treatment. PRO-seq normalized reads for the different RNAi treatments (LacZ and GAF) before and after HS for the plus strand are shown in red, and those for the minus strand are shown in blue. Gene annotations are shown at the *bottom*.

To investigate whether GAF binding is essential for HS activation, we performed PRO-seq in biological replicates of GAF-RNAi-treated cells prior to HS and after 20 min of HS (Spearman's coefficient ranged between 0.96 and 0.99) (Fig. 3A,B; Supplemental Table S1; Supplemental Fig. S1A,B, right panels). The decrease in GAF protein levels after the RNAi treatment produced similar numbers of genes that were significantly activated or repressed by HS (cf. Supplemental Fig. S9A and Fig. 1A); however, a comparison of the HS gene body reads in the GAF-RNAi and LacZ-RNAi control identified many genes that were significantly affected by the knockdown. The HS PRO-seq levels of 20% of activated genes were affected by GAF-RNAi and nearly all were reduced (Fig. 3C, left panel), while <1% of the repressed class were affected (Fig. 3C, right panel), demonstrating that GAF is important for HS activation but not repression. Greater than 90% of the genes that have reduced HS induction after GAF knockdown have GAF binding within ±1 kb of the TSS (Fig. 3C, left panel). Taken together, these results indicate that promoter-bound GAF is indispensable for the proper activation of many HS-activated genes.

GAF is critical for the HS activation of many genes; however, the induction of >70% of the genes that are bound by GAF prior to HS is not affected by GAF knockdown. These two classes of GAF-bound genes, which respond differentially to GAF knockdown, cannot simply be explained by differences in the response to the RNAi treatment, since the GAF ChIP-seq signal for both classes is similarly reduced by the knockdown (Supplemental Fig. S9B). However, GAF-bound genes with GAF-dependent HS activation have significantly higher GAF ChIP-seq intensities when compared with the GAF-bound genes with GAF-independent HS activation (Mann-Whitney *U*-test, *P*-value = 8.96 × 10^−10^) (Supplemental Fig. S9C). The class of GAF-bound, HS-activated genes whose induction is dependent on GAF has a strong preference for GAF binding immediately upstream of the TSS, between −100 and −50 bp (Supplemental Fig. S9D, right panel). Taken together, these results suggest that higher binding levels and positioning upstream and proximal to the TSS are essential for GAF's role in HS activation.

### GAF's role in HS activation correlates with its function in establishing promoter-proximal pausing prior to HS

GAF has been shown to have a role in the establishment of promoter-proximal pausing and consequent HS activation of two classical HSP genes (Glaser et al. 1990; Lee et al. 1992; Lu et al. 1993; O'Brien et al. 1995), and a recent study has demonstrated that pausing was significantly reduced on a large subset of GAF-bound genes upon GAF depletion (Fuda et al. 2015). However, the role of GAF-mediated pausing in gene activation has not yet been studied in a comprehensive genome-wide manner. We hypothesized that GAF's role in HS activation is connected to its ability to create promoter-proximal pausing prior to HS.

To test this hypothesis, we compared the NHS promoter-proximal PRO-seq reads for the LacZ-RNAi control and GAF-RNAi treatment between the subset of GAF-bound genes whose HS induction is dependent on GAF (GAF-dependent HS activation) and the HS-activated genes whose induction is unaffected by GAF depletion (GAF-independent HS activation). As observed in Figure 3D, there is a substantial reduction in the NHS pausing levels after GAF knockdown for genes with GAF-dependent HS activation, while the NHS pausing levels of the GAF-independent class are largely unaffected. To quantify this effect, we compared the LacZ-RNAi and GAF-RNAi NHS reads in the pausing region for genes with GAF-dependent or GAF-independent HS activation. As observed in Figure 3E, most genes with GAF-dependent HS activation have a reduced number of reads (fold change <1) in the pausing region upon GAF knockdown prior to HS. In contrast, the distribution of fold changes for the GAF-independent class is centered around one, indicating that GAF binding prior to HS is not essential to establish pausing at these genes (Mann-Whitney *U*-test, *P*-value < 2.2 × 10^−16^). Figure 3F shows an example of a HS-activated gene that displays GAF-dependent pausing prior to HS whose induction is inhibited by GAF knockdown. Taken together, these results indicate that GAF's role in HS activation strongly correlates with its function in establishing promoter-proximal pausing prior to HS.

Figure 3D also shows that GAF-dependent genes have higher levels of promoter-proximal pausing prior to HS than the GAF-independent ones. Interestingly, they also have a higher average HS/NHS induction (Mann-Whitney *U*-test, *P*-value = 0.0367) (Supplemental Fig. S9E); however, the distribution of HS/NHS fold changes for these two classes mostly overlap. Additionally, there was no preferential enrichment for classical HSP genes in either class.

### Insulator proteins and M1BP are enriched in the promoter region of HS-activated genes with GAF-independent induction

**Figure 4.**
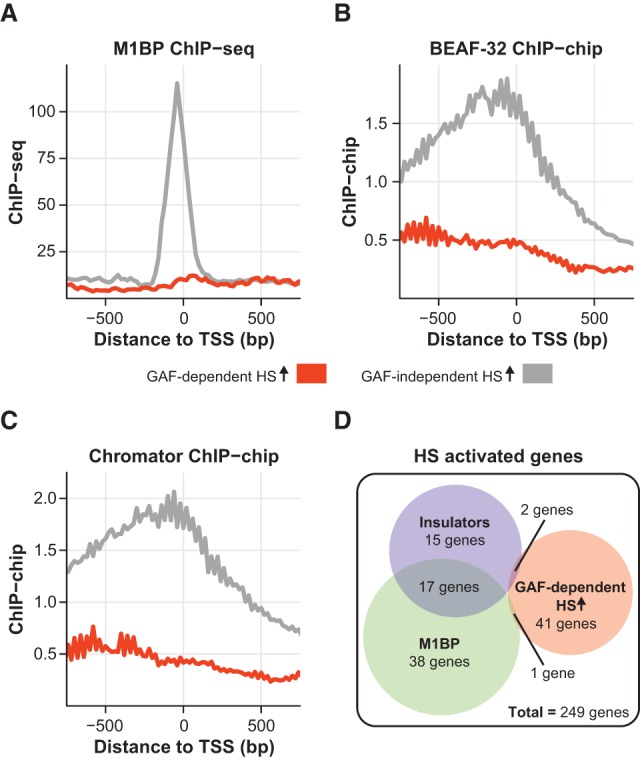
Insulator proteins and M1BP are enriched in the promoter region of HS-activated genes with GAF-independent induction. (*A*–*C*) M1BP ChIP-seq (*A*), BEAF-32 ChIP–chip (BEAF-HB antibody) (*B*), and Chromator ChIP–chip (BR antibody) (*C*) signal between −750 and +750 bp to the TSS (in 20-bp bins) of genes with GAF-dependent (*n* = 44) or GAF-independent (*n* = 199) HS activation (HS ↑). (*D*) Venn diagram showing the overlap between HS-activated genes with GAF-dependent activation and genes bound by M1BP or both insulator proteins (BEAF-32 and Chromator; only genes with insulator binding detected by both antibodies for these two proteins were considered) within ±1 kb of the TSS.

While nearly all activated genes display promoter-proximal pausing prior to HS, we showed that GAF is essential for pausing establishment and HS activation on a subset of these genes. To identify factors that can contribute to the establishment of pausing on GAF-independent genes, we screened modENCODE and other publically available chromatin factor ChIP-seq or ChIP–chip data sets for factors that are differentially enriched in the promoter regions of GAF-independent relative to GAF-dependent genes prior to HS (Supplemental Table S6). Among the factors with the most significant differential enrichment were the transcription factor M1BP (ChIP-seq data from Li and Gilmour 2013), the insulator protein BEAF-32 (ChIP–chip data from Schwartz et al. 2012), and the chromodomain-containing protein Chromator (ChIP–chip data from Kharchenko et al. 2011) (Fig. 4A–C). M1BP is a recently discovered zinc finger transcription factor that has been shown to orchestrate promoter-proximal pausing in a GAF-independent manner (Li and Gilmour 2013). BEAF-32 is one of the insulator-associated proteins identified in *Drosophila* (Zhao et al. 1995), Chromator was initially identified as a mitotic spindle protein and later implicated in the regulation of chromosome structure through partial cooperation with BEAF-32 (Rath et al. 2006; Gan et al. 2011), and both of these proteins are enriched at the boundaries of physical chromosomal domains (Hou et al. 2012; Sexton et al. 2012). The factor with the highest differential enrichment for promoter-bound genes in our screen was the tandem kinase JIL-1 (Supplemental Table S6), which has been shown previously to interact with Chromator (Rath et al. 2006). However, a comparison between the JIL-1 ChIP–chip intensities of genes with GAF-dependent and GAF-independent HS activation did not show the same striking differences that were observed for M1BP, BEAF-32, and Chromator (Supplemental Fig. S10). Remarkably, almost no overlap exists between genes with GAF-dependent HS activation and genes bound by M1BP or insulator proteins within ±1 kb of the TSS (Fig. 4D). The mutually exclusive distributions of GAF and M1BP in promoter-proximal pausing has been reported previously (Li and Gilmour 2013), and our results suggest a possible role for M1BP in pausing and HS activation. Similarly to M1BP, the mutually exclusive distribution of insulator proteins and the GAF-dependent subset suggests that BEAF-32 and/or Chromator may have a role in generating promoter-proximal pausing when bound proximally to the TSS. GAF has also been classified as an insulator protein with enhancer-blocking activity (Ohtsuki and Levine 1998; Schweinsberg et al. 2004), which suggests a possible overlap between insulator function and a role in maintaining an open chromatin environment that enables promoter-proximal pausing and opens the possibility for a novel role of BEAF-32 and Chromator as pausing factors. Another possible explanation is that these insulator proteins reside between GAF and the TSS, therefore blocking any activity of GAF on the promoter, which could explain why pausing is not affected by GAF depletion at insulator-bound promoters.

### M1BP is important for promoter-proximal pausing and HS activation of a subset of M1BP-bound HS-activated genes

**Figure 5.**
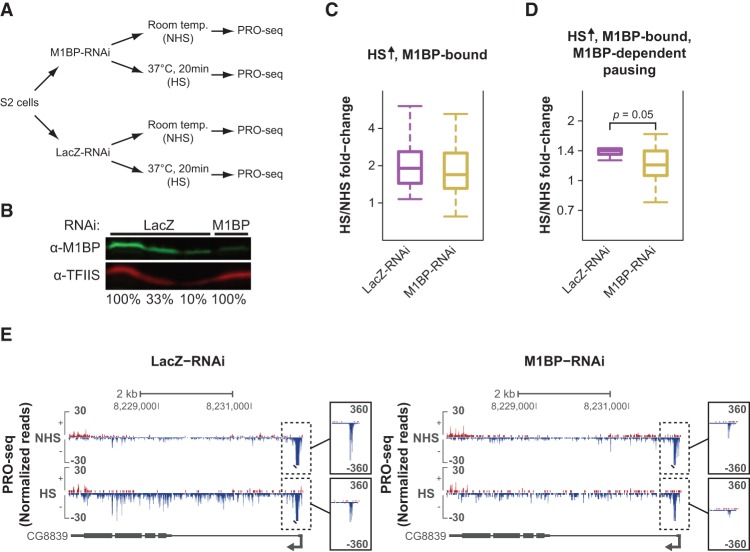
M1BP is important for pausing and HS activation of a subset of M1BP-bound genes with GAF-independent induction. (*A*) Experimental setup. *Drosophila* S2 cells were treated with either M1BP-RNAi or LacZ-RNAi for 5 d. Nuclei were then isolated for PRO-seq after cells were incubated at room temperature (NHS) or heat-shocked for 20 min (HS). (*B*) Western blot of whole-cell extracts from LacZ-RNAi-treated and M1BP-RNAi-treated cells using antibodies detecting M1BP and TFIIS (loading control). One-hundred percent is equivalent to 1.5 × 10^6^ cells. (*C*) Box plot showing the HS/NHS fold change of M1BP-RNAi or LacZ-RNAi control cells for all M1BP-bound, HS-activated genes (HS ↑). (*D*) Box plot showing the HS/NHS fold change of M1BP-RNAi or LacZ-RNAi control cells for M1BP-bound, HS-activated genes with M1BP-dependent pausing. Mann-Whitney *U*-test, *P*-value = 0.05. (*E*) Representative view in the UCSC genome browser (Kent et al. 2002) of a gene with M1BP-dependent pausing prior to HS whose activation is decreased by M1BP-RNAi treatment. PRO-seq normalized reads for the different RNAi treatments (LacZ and M1BP) before and after HS for the plus strand are shown in red, and those for the minus strand are shown in blue. Gene annotations are shown at the *bottom*.

To investigate whether M1BP has a role in pausing and HS activation of M1BP-bound genes with GAF-independent HS activation, we performed PRO-seq in biological replicates of M1BP-RNAi-treated cells prior to HS and after 20 min of HS (Spearman's coefficient ranged between 0.97 and 0.99) (Fig. 5A,B; Supplemental Table S1; Supplemental Fig. S11A,B, right panels). As seen in Figure 5C, M1BP depletion by RNAi has a small effect on the HS activation of M1BP-bound, HS-activated genes when compared with the LacZ-RNAi control; however, this effect was not statistically significant. To assess whether, like GAF, M1BP's role in HS activation is associated with its role in establishing promoter-proximal pausing, we then focused on the subset of HS-activated, M1BP-bound genes that display M1BP-dependent pausing prior to HS. As seen in Figure 5D, M1BP knockdown has a significant effect on the HS activation of this subset of genes (Mann-Whitney *U*-test, *P*-value = 0.05), indicating that M1BP has a role in pausing establishment and HS activation of at least a subset of M1BP-bound genes. Figure 5E shows an example of a gene that displays M1BP-dependent pausing prior to HS whose HS induction is affected by M1BP knockdown. Thus, M1BP, like GAF, is important for pausing and HS activation of a subset of genes, supporting the hypothesis that pausing is a prerequisite for HS activation.

### HSF is essential for the induction of only a small minority of HS-activated genes

**Figure 6.**
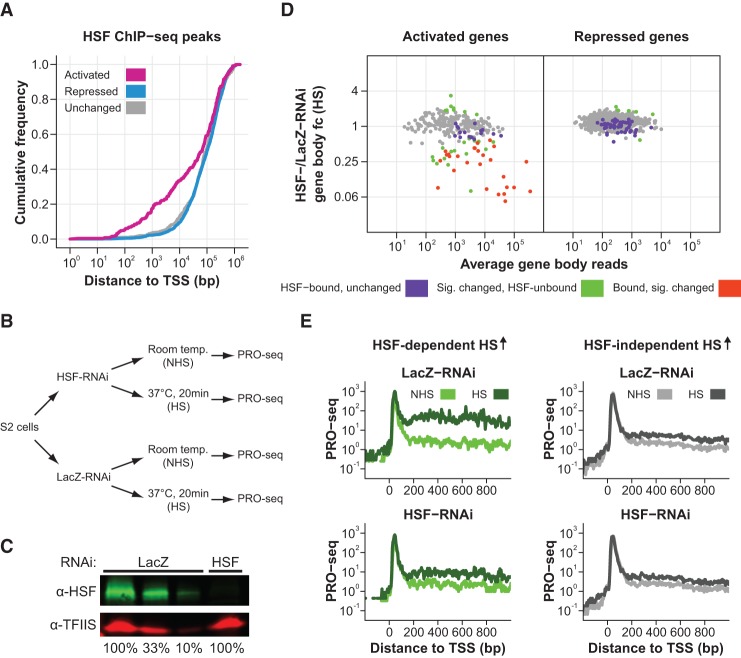
HSF is essential for the induction of only a small minority of HS-activated genes and activates genes by stimulating the release of paused Pol II. (*A*) Cumulative distribution plots of the distance between the closest HSF ChIP-seq peak and the TSS of each gene in the HS-activated (*n* = 249), HS-repressed (*n* = 2300), and unchanged (*n* = 517) classes. (*B*) Experimental setup. *Drosophila* S2 cells were treated with either HSF-RNAi or LacZ-RNAi for 5 d. Nuclei were then isolated for PRO-seq after cells were incubated at room temperature (NHS) or heat-shocked for 20 min (HS). (*C*) Western blot of whole-cell extracts from LacZ-RNAi-treated and HSF-RNAi-treated cells using antibodies detecting HSF and TFIIS (loading control). One-hundred percent is equivalent to 1.5 × 10^6^ cells. (*D*) DESeq2 analysis to determine the effect of HSF-RNAi treatment on the PRO-seq gene body reads after HS for the HS-activated and HS-repressed classes. We used DESeq2 to identify significantly changed genes between HSF-RNAi HS and LacZ-RNAi HS cells, and the results are displayed as MA plots. Significantly changed genes were defined using an FDR of 0.001. HSF-bound genes are labeled in purple, significantly changed genes (according to DESeq2) are labeled in green, and genes that are both HSF-bound and significantly changed are labeled in orange. (fc) Fold change. (E) PRO-seq read density between −200 and +1000 bp to the TSS (in 5-bp bins) of genes with HSF-dependent (*n* = 44) or HSF-independent (*n* = 197) HS activation (HS ↑) for the LacZ-RNAi and HSF-RNAi data sets before (NHS) and after HS.

HSF is the evolutionarily conserved master regulator of the HS response and is essential for the activation of classical HSP genes (Wu 1995). Inducible HSF binding at those genes is critical for the recruitment of the positive elongation factor P-TEFb (Lis et al. 2000), which modulates the release of Pol II into productive elongation. We used our previously published HSF ChIP-seq data sets performed before and after 20 min of HS induction (Guertin and Lis 2010) to determine whether HSF also preferentially binds to noncanonical HS-activated genes. HSF ChIP-seq peaks are closer to the TSS in the HS-activated class when compared with the HS-repressed (Kolmogorov-Smirnov test, *P*-value = 4.04 × 10^−12^) (Fig. 6A) and unchanged (Kolmogorov-Smirnov test, *P*-value = 1.6 × 10^−7^) gene classes (Fig. 6A). Surprisingly, even though HSF is enriched in the proximity of activated genes, <20% of those genes have an HSF ChIP-seq peak within ±1 kb of the TSS. The existence of HSF-independent genes has been demonstrated previously in *Drosophila* (Gonsalves et al. 2011). However, our study substantially expands the number of identified genes and offers a more comprehensive view of HSF-independent regulation due to the considerably higher resolution and sensitivity afforded by our binding and nascent transcription assays. These results indicate that HSF can activate genes when bound to distal enhancer sites or that there are other factors dictating the induction of HS-activated genes.

### HSF activates genes by stimulating the release of paused Pol II

To investigate HSF's roles during the HS-induced transcriptional response, we performed PRO-seq in biological replicates of HSF-RNAi-treated cells prior to HS and after 20 min of HS (Spearman's coefficient ranged between 0.96 and 0.99) (Fig. 6B,C; Supplemental Table S1; Supplemental Fig. S1A,B, middle panels). Comparison of the HS gene body reads in the LacZ-RNAi control and HSF-RNAi for activated and repressed genes shows that the HS PRO-seq levels of 20% of activated genes were affected by HSF-RNAi, while a significant change was observed for only <1% of the repressed class, demonstrating that HSF is important for HS activation but not repression (Fig. 6D).

Most of the activated genes that have HSF binding within ±1 kb of the TSS have reduced HS induction after HSF knockdown (Fig. 6D, left panel, orange points). HSF-bound genes with compromised induction upon HSF knockdown are enriched for HSF binding immediately upstream (within 200 bp) of the TSS, while the unaffected class displays a random distribution of distances (Supplemental Fig. S12A). Furthermore, HSF-bound genes with reduced HS induction have significantly higher HSF ChIP-seq binding intensity when compared with the unaffected class (Mann-Whitney *U*-test, *P*-value = 2.5 × 10^−3^) (Supplemental Fig. S12B), indicating that higher HSF binding levels and positioning upstream and proximal to the TSS are important for the induction of HSF's target genes. Additionally, a comparison of all induced, HSF-dependent genes with the remainder of HS-activated genes (HSF-independent HS activation) showed that genes depending on HSF have stronger HS induction (Fig. 6E). As expected, HSF is essential for the HS activation of all seven classical HSP genes in our gene list, which strongly contributes to the higher HS induction of HSF-dependent genes relative to genes with HSF-independent HS activation (Fig. 6E).

HSF depletion does not affect the induction of most genes that are not bound by HSF within ±1 kb of the TSS (Fig. 6D, left panel, gray points). Nonetheless, the presence of significantly changed genes with no proximal HSF binding (Fig. 6D, left panel, green points) indicates that HSF may be able to mediate activation at distal enhancer sites on a small subset of genes. The enhancer activity of HSF has been shown previously in a focused study of *Hsp70* to be weak and require large arrays of HSF-binding sites (Bienz and Pelham 1986). This early study and the rarity with which we found HSF acting at a distance might be explained if such long-range interactions required specialized binding sites and chromatin architecture. Clearly, the preferred mode of HSF action is close to promoters.

Composite profiles show that the average pausing levels of genes with HSF-dependent and HSF-independent HS activation are not affected by HS in both LacZ-RNAi and HSF-RNAi conditions (Fig. 6E), indicating that HSF depletion and HS do not have much of an effect on pausing. Quantification of the effect of HSF knockdown on pausing levels in both NHS and HS conditions for all HS-activated genes revealed that the pausing level of only one gene was significantly affected by the knockdown (Supplemental Fig. S12C). Taken together, these results suggest that HSF acts mainly at the release of paused Pol II into productive elongation, which is consistent with the critical role of HSF in the recruitment of the pause release factor P-TEFb to *Hsp70* (Lis et al. 2000).

### HS transcriptional repression results in a decrease of promoter-proximally paused Pol II

**Figure 7.**
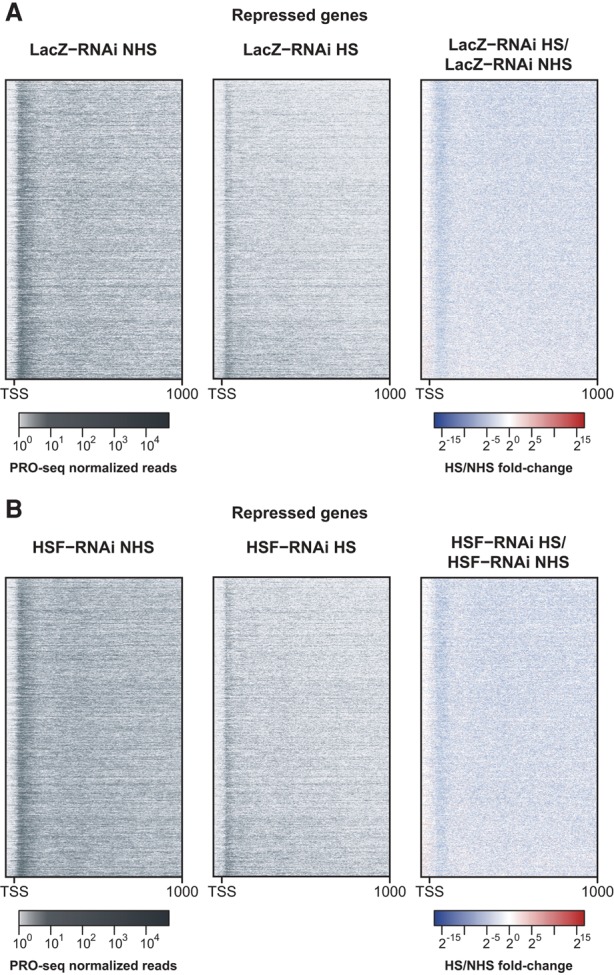
HS transcriptional repression is HSF-independent and results in a decrease of promoter-proximally paused Pol II. Heat maps showing the NHS PRO-seq levels (*left* panel), the HS PRO-seq levels (*middle* panel), and the fold change between the two conditions (*right* panel) between −50 and +1000 bp to the TSS (in 5-bp bins) of HS-repressed genes (*n* = 2300) for the LacZ-RNAi (*A*) and HSF-RNAi (*B*) treatments. Genes in both *A* and *B* are sorted by the HS/NHS PRO-seq fold change in the LacZ-RNAi condition (highest to lowest).

Our data revealed that HS induction causes a vast transcriptional shutdown, with thousands of genes being repressed after 20 min of increased temperatures (Fig. 1A). To elucidate the mechanisms involved in this repression, we observed the PRO-seq profile for all repressed genes plotted as heat maps before and after HS (Fig. 7A). This analysis indicates the presence of enriched PRO-seq reads in the region immediately downstream from the TSS, representing promoter-proximally paused polymerases. Both gene body reads and reads in this promoter-proximal region are reduced after HS, which is also evidenced by the overall blue color of the fold change heat map (Fig. 7A, right panel). The overall NHS and HS distributions and the HS/NHS fold change are very similar after HSF depletion by knockdown (Fig. 7B), indicating that HSF does not play a role in gene repression by HS.

## Discussion

In this study, we used PRO-seq to comprehensively characterize the direct changes in Pol II distribution that occur in *Drosophila* S2 cells in the minutes following HS. We show that the HS response is more general than previously appreciated, with thousands of genes being repressed and hundreds activated by heat. This latter class is not limited to the group of cellular chaperones that are known to be activated by stress (Lindquist and Craig 1988) and includes hundreds of other genes with various cellular functions (Supplemental Table S2). Surprisingly, only 20% of the activated genes are regulated by HSF, which was previously believed to be the major orchestrator of the response. Moreover, we show that promoter-proximal pausing is highly pronounced and prevalent among activated genes prior to HS. GAF, which has been shown to be important for establishing pausing, is highly enriched at the promoters of HS-activated genes, and our results suggest that GAF-mediated pausing in a subset of these genes is essential for HS activation. Furthermore, our results indicate that HS activation of HSF-dependent genes is regulated at the level of pausing release, whereas HS repression of thousands of genes is regulated at the step of transcription initiation in *Drosophila*, and this process is independent of HSF. Very recently, we showed in mice that HS activation is similarly regulated by HSF at the level of pause release; however, in contrast to *Drosophila*, HS repression of genes is also mediated at pause release (Mahat et al. 2016). In both mammals and *Drosophila*, the widespread transcriptional repression is independent of HSF. Overall, by measuring how transcription changes after HS, our results provide insights into mechanisms of transcription activation and repression, the key regulating factors, and the steps in the transcription cycle that are modulated.

### GAF-mediated promoter-proximal pausing is essential for the HS activation of a subset of genes

Classical HSP genes accumulate paused Pol II molecules between 20 and 50 bp downstream from the TSS prior to HS (Rougvie and Lis 1988; Rasmussen and Lis 1993). Our results and analyses greatly expand on these previous findings and indicate that pausing is a common feature among HS-activated genes and is not specific to the highly induced class of molecular chaperones. Previous studies have shown that the paused Pol II complex on *Hsp70* and many other genes is remarkably stable (Henriques et al. 2013; Buckley et al. 2014; Jonkers et al. 2014), and this stably paused molecule can help to maintain an open chromatin environment that is accessible to transcription factors that will promote the release of Pol II into productive elongation, mediating a rapid response to HS. The open chromatin state of our newly identified HS-activated genes is confirmed with the higher DNase I hypersensitivity signal observed in the promoter region relative to repressed and unchanged genes (Supplemental Fig. S7A).

GAF has been shown previously to be important for establishing pausing and is highly enriched in the promoter region of activated genes prior to HS. GAF is essential for HS activation of a subset of GAF-bound genes that have high levels of GAF binding in the region immediately upstream of the core promoter, indicating that GAF's positioning and levels are important for its role in the HS response. We also observed that the pausing levels of genes with GAF-dependent HS activation are dramatically reduced upon GAF depletion prior to HS. Transgenic studies of the model *Hsp70* gene have demonstrated that the presence of the GAF-binding element is essential for generating pausing at this gene and that pausing level changes created by mutating the core promoter strongly correlate with the promoter's potential to induce transcription upon HS induction (Lee et al. 1992). Our results expand on these studies and demonstrate that GAF depletion prior to HS in the native chromatin environment of a subset of HS-activated genes abrogates Pol II pausing levels and the consequent induction of these genes by HS. Importantly, other GAF-bound genes that maintain pausing upon GAF knockdown due to the activity of other pausing factors like M1BP and possibly the insulator proteins BEAF-32 and Chromator remain fully HS-inducible. We propose a model in which GAF-mediated pausing is essential to maintain an open chromatin environment at the promoter region prior to HS (Supplemental Fig. S13A). When GAF is depleted by knockdown and pausing is not properly established, the promoter loses its potential to induce transcription after HS (Supplemental Fig. S13A).

### HSF acts at the step of promoter-proximal pausing release

HSF depletion has almost no effect on pausing reads both before and after HS (Supplemental Fig. S12C), and the average pausing levels are largely unaffected by HS and HSF knockdown (Fig. 6E). The amount of pausing is determined by the transcription recruitment/initiation rate and the rate of escape into productive elongation. If HSF was acting at the step of Pol II initiation, we would expect the pausing levels to be reduced upon HSF depletion, which is not observed. Therefore, we propose a model in which, after being recruited to the promoter region upon HS, HSF promotes the release of Pol II into the gene body (Supplemental Fig. S13B), likely through the indirect recruitment of P-TEFb, which has been shown to be the case for classical HSP genes (Lis et al. 2000). Pausing also maintains an open chromatin environment that is accessible to transcription activators such as HSF. In this model, the activity of factors that are important for establishing pausing prior to HS, such as GAF, is crucial for HSF-dependent HS activation (Supplemental Fig. S13B), and failure to generate pausing prevents the induction of HSF target genes. Less than 20% of the genes with HSF-dependent HS activation are also dependent on GAF for activation, indicating that the action of other factors such as M1BP and possibly BEAF-32 and Chromator is important for pausing and consequent HS activation of these HSF-dependent genes.

### HS causes a rapid and broad reduction in transcription, which is regulated at the transcription initiation step and is independent of HSF

Early low-resolution studies in *Drosophila* polytene chromosomes have shown that HS causes a genome-wide down-regulation of transcription (Spradling et al. 1975; Jamrich et al. 1977), presumably to reduce the accumulation of misfolded protein aggregates. Although this has been a paradigm in the HS field, higher-resolution genome-wide studies have failed to identify all of the primary genes that are repressed by heat, mostly due to the limitations of measuring steady-state levels of mRNA, which requires that the mRNAs already present in the cells have shorter half-lives than the HS time points used in the experiment. Our results provide definitive evidence to support the widespread shutdown of transcription caused by HS. We identified and quantified the genes with significantly reduced transcription and demonstrated that the HS-repressive response is very rapid, with more than a thousand genes being repressed after only 5 min of HS (Supplemental Fig. S6A). Furthermore, the Pol II density in the promoter-proximal region, which represents the paused Pol II molecules, is also significantly reduced across all HS-repressed genes (Fig. 7). The accumulation of Pol II in the pausing region depends on both the transcription initiation rate and the rate of escape into productive elongation. The reduction in pausing levels thus indicates that the recruitment and initiation of Pol II are affected by HS (Supplemental Fig. S13C).

The HS-induced binding of HSF is not essential for the genome-wide transcriptional repression (Fig. 7), and, given the magnitude of this repressive response, we believe that it is unlikely that one single transcription repressor is responsible for inhibiting transcription initiation in all HS-repressed genes. We considered three possible mechanisms, which are not mutually exclusive, that could be responsible for HS-mediated repression. (1) The activity of a general transcription factor that is involved in recruitment of Pol II to the promoter could be modulated by heat stimulus. (2) Changes in nucleosomal composition or positioning induced by heat could generate an unfavorable chromatin environment that would prevent transcription initiation and elongation. A previous study has demonstrated that HS results in decreased nucleosome turnover genome-wide within gene bodies; however, the same pattern was observed after drug inhibition of Pol II elongation, arguing that reduced nucleosome turnover may be a consequence rather than the cause of the genome-wide transcriptional repression (Teves and Henikoff 2011). (3) A genome-wide rearrangement of the three-dimensional (3D) chromatin structure could either disrupt long-range interactions that are needed for transcription or allow new long-range interactions that repress transcription initiation, which is supported by a recent study in a different *Drosophila* cell line that demonstrated that HS induces a genome-wide rearrangement in the 3D nuclear architecture (Li et al. 2015). Any model must accommodate our new observations that (1) recruitment of Pol II is the step in the transcription cycle that is regulated, (2) HSF is not involved in the repression, and (3) the specifically repressed genes identified here and their level of down-regulation must be accommodated by any proposed regulatory factor interactions.

## Materials and methods

### GAF-RNAi, HSF-RNAi, M1BP-RNAi, and LacZ-RNAi treatments

*Drosophila* S2 cells were grown in M3 + BPYE medium supplemented with 10% FBS until they reached 3 × 10^6^ to 5 × 10^6^ cells per milliliter. At this point, the cells were split into 1 × 10^6^ cells per milliliter in serum-free M3 + BPYE medium, and the desired volume of cells was mixed with LacZ, GAF, HSF, or M1BP dsRNA to a final concentration of 10 µg/mL. After incubation for 45 min at 25°C, an equal volume of M3 + BPYE medium supplemented with 20% FBS was added to the cells. After 2.5 d, the cells were split 1:2 into two new flasks, and more dsRNA was added to keep the final concentration at 10 µg/mL. After 2.5 d, the cells were HS-treated and harvested for nuclei isolation. The M1BP-RNAi treatment was performed separately with its own LacZ control.

The dsRNAs used in these experiments were transcribed from a dsDNA template that had a T7 polymerase promoter at both ends. The DNA templates were generated by PCR using the following primers: LacZ forward (GAATTAATACGACTCACTATAGGGAGAGATATCCTGCTGATGAAGC), LacZ reverse (GAATTAATACGACTCACTATAGGGAGAGCAGGAGCTCGTTATCGC), GAF forward (GAATTAATACGACTCACTATAGGGATGGTTATGTTGGCTGGCGTCAA), GAF reverse (GAATTAATACGACTCACTATAGGGATCTTTACGCGTGGTTTGCGT), HSF forward (GAATTAATACGACTCACTATAGGGAGAGCCTTCCAGGAGAATGCA), HSF reverse (GAATTAATACGACTCACTATAGGGAGAGCTCGTGGATAACCGGTC), M1BP forward (from Li and Gilmour 2013) (GAATTAATACGACTCACTATAGGGAGAGCAGCCAAATTGCTTGTCC), and M1BP reverse (from Li and Gilmour 2013) (GAATTAATACGACTCACTATAGGGAGAAGACGGTGAAGACGCCC).

### Western blot analysis to assess knockdown levels

Western blots were performed using standard conditions, and dilutions of the LacZ-RNAi control samples were used as a quantitative indication of signal linearity. Laboratory stocks of rabbit anti-HSF and anti-GAF antibodies and guinea pig anti-TFIIS antibody were used at dilutions of 1:2000, 1:500, and 1:3000, respectively. The rabbit anti-M1BP antibody was provided by David Gilmour's laboratory and was used at a 1:5000 dilution. We used 1 mg/mL IRDye 800CW donkey anti-rabbit and 1 mg/mL IRDye 680LT donkey anti-guinea pig as secondary antibodies at a 1:15,000 dilution, and the membrane was imaged using the LI-COR Odyssey imaging system.

### HS treatments

For the HS treatments, an equal volume of M3 + BPYE medium (no serum) at 48°C was added to the cells, and the cultures were incubated for the desired time at 37°C.

### Preparation of PRO-seq libraries

Nuclei isolation and PRO-seq library preparation were performed as described previously (Kwak et al. 2013).

### Preparation of RNA-seq libraries

Total RNA from S2 cells was extracted using TRIzol reagent (Thermo Fisher Scientific) and then isolated from the aqueous phase using the EZNA total RNA kit I (Omega Bio-tek). The following steps were performed by the Cornell RNA Sequencing Core (Department of Biomedical Sciences, College of Veterinary Medicine, Cornell University). Poly(A)^+^ RNA was isolated with the NEBNext Poly(A) mRNA magnetic isolation module (New England Biolabs). TruSeq-barcoded RNA-seq libraries were generated with the NEBNext Ultra Directional RNA library preparation kit (New England Biolabs). Each library was quantified with a Qubit 2.0 (dsDNA HS kit, Thermo Fisher Scientific), and the size distribution was determined with a fragment analyzer (Advanced Analytical) prior to pooling.

### PRO-seq data acquisition

PRO-seq libraries were sequenced in 50-nucleotide (nt) runs on the Illumina HiSeq using standard protocols at the Cornell Biotechnology Resource Center (http://www.BRC.cornell.edu). Raw sequencing reads were processed using the FastX toolkit (http://hannonlab.cshl.edu/fastx_toolkit/index.html). Illumina adapters were removed with the fastx_clipper tool, and reads were trimmed to 26-mers using fastx_trimmer. Sequencing reads <15 nt were discarded. fastx_reverse_complement was then used to generate the reverse complement of the sequencing reads, which correspond to the sense strand of nascent RNA in the nucleus. Reads were aligned uniquely to the *D. melanogaster* dm3 reference genome using Bowtie (Langmead et al. 2009) with up to two mismatches. Histograms of the 3′ end position of each mapped read in base-pair resolution were generated in bedgraph format and used for all subsequent analyses. Supplemental Table S1 contains a summary of sequencing yields and the number of reads that mapped uniquely to the genome or other annotations. Replicates were highly correlated and pooled for further analyses (Supplemental Fig. S1). Sequencing data sets were deposited at Gene Expression Omnibus (GEO) under accession number GSE77607.

### PRO-seq normalization method

We used a previously published Pol II ChIP-seq data set in *Drosophila* S2 cells (Teves and Henikoff 2011) to identify genes whose transcription does not change during HS. Unlike ChIP-seq reads, which can originate from both sense and antisense strands, PRO-seq reads are strand-specific. Therefore, in order to increase the likelihood of selecting genes that have the majority of their reads originating from the sense strand, we used our PRO-seq LacZ-RNAi control data sets (NHS and 20 min of HS) to identify and filter out genes that have high levels of transcription in the antisense strand. To identify those genes, we calculated the fraction of PRO-seq reads originated from the antisense strand for each gene and kept only the ones whose fraction was ≤0.2 for both the NHS and 20-min HS conditions. Because of the high background in ChIP-seq data, we then focused on genes with the highest levels of Ser2-P ChIP signal (*Z*-score > 3) (Core et al. 2012), assuming that these contained the highest densities of transcribing Pol II over background. In order to obtain a final subset of unaffected genes, we filtered out the ones whose gene body fold change between NHS and HS conditions was <0.85 and >1.15, resulting in 335 genes. The mRNA levels of this subset of genes were also unaffected after HS (Supplemental Fig. S2), which is consistent with the transcription levels of these genes not changing after induction.

We then used the sum of the total number of gene body reads for all 335 genes to generate normalization factors in our PRO-seq data to normalize the data sets between replicates and different time points. Since the GAF-RNAi, HSF-RNAi, and M1BP-RNAi treatments did not result in genome-wide changes in transcription in both the NHS and 20-min HS time points, we used the same subset of 335 unaffected genes to normalize the data sets between different RNAi treatments (LacZ, GAF, HSF, and M1BP).

To assess the efficacy of this normalization method, we examined the correlation between gene body and promoter reads for replicates (Supplemental Fig. S1) and gene body reads across different time points (Supplemental Fig. S3A,B). All replicates showed good correlation and time points that were closer to each other have higher correlation coefficients and better fits to the 1:1 diagonal. Moreover, for the RNAi treatments, we examined the correlation between gene body reads across different conditions (NHS and 20-min HS) and observed that the NHS data sets have higher correlation coefficients and better fits to the 1:1 diagonal when plotted against each other, and the same was observed for the HS treatments (Supplemental Fig. S3C,D). Taken together, these results indicate that the normalization method worked appropriately.

### Differential expression analysis using DESeq2

We used DESeq2 (Love et al. 2014) to identify genes whose gene body reads significantly change after HS, starting with a list of 9452 nonoverlapping genes described previously (Core et al. 2012). Gene body reads were collected from 200 bp downstream from the TSS, and we used different 3′ limits for each time point, assuming a conservative estimate for Pol II transcription elongation rate of 1 kb/min. We provided our own normalization factors for the DESeq2 calculations, which were obtained as described above. We used an FDR of 0.001 to identify activated and repressed genes. Unchanged genes were defined as having an adjusted *P*-value of >0.5 and log_2_ fold change higher than −0.25 and less than 0.25.

### Upstream transcription filter

To minimize the number of false positives caused by changes in run-through transcription originated at the upstream gene, we implemented a filter to exclude from our analyses genes that have high levels of transcription in the region immediately upstream of the TSS. For each gene, we obtained the read counts from a window upstream of the TSS (−500 to −100 bp of the TSS) and a window in the gene body (+300 to +700 bp of the TSS) (Supplemental Fig. S4A). The 3′ limit of the upstream window was defined as −100 bp to the TSS to avoid confounding effects of potentially misannotated TSSs. In the case of the gene body window, the 5′ limit was defined as +300 bp to the TSS to avoid the region immediately downstream from the TSS, which can contain peaks of promoter-proximal paused Pol II. We then took the ratio of the read counts in these two regions for each gene, taking into consideration the number of mappable positions in the two windows (Supplemental Fig. S4A). This ratio was named the “upstream ratio” and was later used to exclude false positive genes from our analyses.

In order to verify whether we could distinguish true and false positives based on the upstream ratio and define the appropriate cutoff to filter out false positive genes, we visually inspected 100 randomly selected HS-activated genes and classified each one as true or false positive based on the presence or absence of run-through transcription. The average mRNA levels (Supplemental Fig. S4B) are higher for the genes that were defined as true positives, which provides an independent verification of the criteria that were used to define true and false positives.

The distribution of upstream ratios for the LacZ-RNAi HS condition was very distinct for true and false positives, with very little overlap (Supplemental Fig. S4C), indicating that this metric could be used to identify false positives. In order to define the optimal cutoff, we evaluated the performance of all potential cutoffs from 0 to 1 in 0.01 increments and used the accuracy metric (true positives + true negatives)/total to identify the cutoff with the best performance (Supplemental Fig. S4D). The horizontal line in Supplemental Fig. S4C represents the chosen cutoff (0.23). Filtering out genes with upstream ratios >0.23 eliminated all but one false positive, with only a minor loss of true positive genes.

We then filtered out genes with upstream ratios >0.23 in the HS-activated and HS-repressed classes to generate the final subsets of genes that were used in all subsequent analyses. In order to use the condition with the highest PRO-seq levels for upstream ratio calculation, we used the NHS upstream ratio to filter the repressed subsets of genes and the HS upstream ratio to filter the activated subsets for every HS and RNAi treatment.

### GO analysis

GO analysis on HS-activated and HS-repressed genes was performed using the Functional Annotation tool from DAVID (Huang et al. 2009), in which “GOTERM_BP_FAT” was selected.

### Composite profiles

The composite profiles in Figures 2, A and C; 3D; 4, A–C; and 6E and Supplemental Figures S7A and S10 represent the median from 1000 subsamplings of 10% of the genes in each class, as described previously (Core et al. 2012; Danko et al. 2013). The shaded areas in Figure 2, A and C, and Supplemental Fig. S7A represent the 75% confidence intervals.

### Promoter-proximal pausing analysis

The “pausing region” was defined as the 50-bp interval with the highest number of reads within −50 to +150 bp of the TSS. This region was defined using the LacZ-RNAi control NHS condition, and the same interval was used for all of the other treatments and conditions. The pausing index was then calculated as the ratio of the read density in the pausing region (reads/mappable bases) and the read density in the gene body (as defined above). Genes were classified as paused as described previously (Core et al. 2008). We used DESeq2 to identify genes whose pausing levels significantly change after HS using an FDR of 0.001.

### Transcription factor binding analysis

We used bedtools closest (Quinlan and Hall 2010) to identify the closest HSF, GAF, or M1BP ChIP-seq peak to the TSS of every transcription unit in our list. HSF-, GAF-, or M1BP-bound genes were defined as having a ChIP-seq peak within ±1000 bp of the TSS. For the modENCODE factors, bound genes were defined as having a “regions_of_sig_enrichment” (from ChIP–chip gff3 file) within ±1000 bp of the TSS.

### De novo motif search

De novo motif analysis of the promoter regions of HS-activated genes (−300 to +50 bp of the TSS) was performed using MEME (Bailey and Elkan 1994). Individual matches to the HSE's position weight matrix were identified by FIMO (Grant et al. 2011).

### RNA-seq data acquisition and analysis

RNA-seq libraries were sequenced in 100-nt runs on the Illumina HiSeq using standard protocols at the Cornell Biotechnology Resource Center (http://www.BRC.cornell.edu). Illumina adapters were removed with the fastx_clipper tool (http://hannonlab.cshl.edu/fastx_toolkit/index.html), and sequencing reads <20 nt were discarded. Reads were aligned to the *D. melanogaster* dm3 reference genome/transcriptome using TopHat2 (Kim et al. 2013) with the following parameters: “--library-type fr-firststrand --no-novel-juncs.” Supplemental Table S3 contains a summary of sequencing yields and the number of reads that mapped to the genome/transcriptome or other annotations. Sequencing data sets are available under GEO accession number GSE77607.

FPKM (fragments per kilobase per million mapped fragments) values for each gene were generated with Cuffnorm (Trapnell et al. 2010) using the BAM files generated by TopHat2 as input. Raw counts for each gene were obtained using HTSeq-count (Anders et al. 2014) and used as input for differential expression analysis using DESeq2 (Love et al. 2014). We used an FDR of 0.001 to identify genes whose mRNA levels significantly increase or decrease upon HS.

## Supplementary Material

Supplemental Material
