# Supplementary material for: Transcription factors GAF and HSF act at distinct regulatory steps to modulate stress-induced gene activation
Source: Genes Dev. 2016 Aug 1;30(15):1731–46. doi: 10.1101/gad.284430.116 (PMC5002978; doi:10.1101/gad.284430.116)
Supplement: Supplemental Material [file supp_30_15_1731__index.html]

Transcription factors GAF and HSF act at distinct regulatory steps to modulate stress-induced gene activation — Supplemental Material 

# Transcription factors GAF and HSF act at distinct regulatory steps to modulate stress-induced gene activation

## Supplemental Material

- Supplemental\_FigureS1.pdf
- Supplemental\_FigureS8.pdf
- Supplemental\_FigureS12.pdf
- Supplemental\_TableS6.xlsx
- Supplemental\_FigureS6.pdf
- Supplemental\_FigureS10.pdf
- Supplemental\_TableS4.xlsx
- Supplemental\_FigureS4.pdf
- Supplemental\_TableS2.xlsx
- Supplemental\_FigureS2.pdf
- Supplemental\_FigureS9.pdf
- Supplemental\_FigureS13.pdf
- Supplemental\_FigureS7.pdf
- Supplemental\_FigureS11.pdf
- Supplemental\_TableS5.xlsx
- Supplemental\_FigureS5.pdf
- Supplemental\_TableS3.pdf
- Supplemental\_FigureS3.pdf
- Supplemental\_TableS1.pdf
