# Supplementary material for: Transcription factors GAF and HSF act at distinct regulatory steps to modulate stress-induced gene activation
Source: Genes Dev. 2016 Aug 1;30(15):1731–46. doi: 10.1101/gad.284430.116 (PMC5002978; doi:10.1101/gad.284430.116)
Supplement: Supplemental Material [file supp_gad.284430.116_Supplemental_FigureS1.pdf]

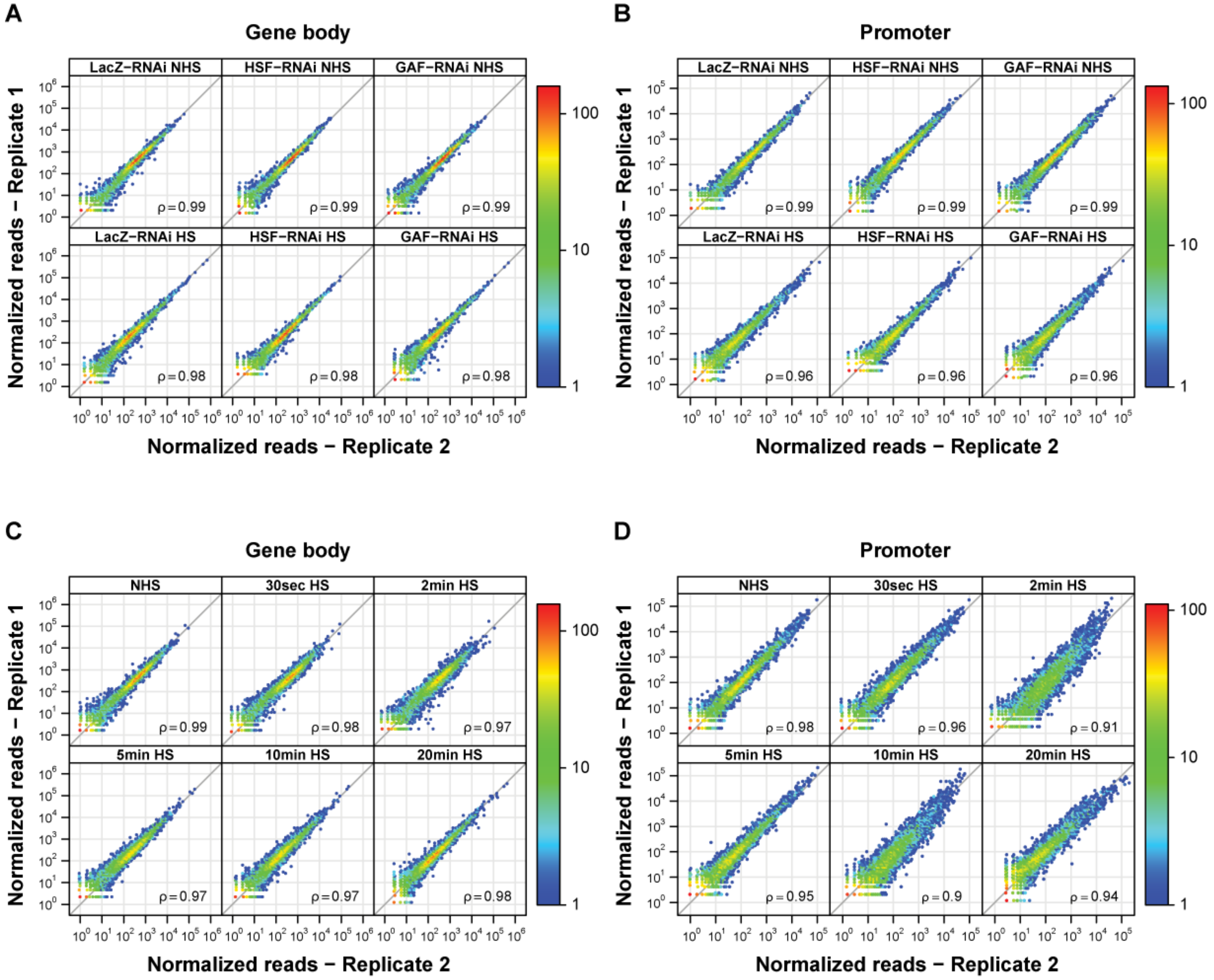

Figure S1: Biological replicates of PRO-seq libraries were highly correlated for both promoter and gene body regions. (A, B) Correlation plots between PRO-seq reads of biological replicates for the different RNAi treatments (LacZ, HSF and GAF) in **(A)** gene body (200 bp downstream of the TSS to the polyadenylation site) and **(B)** promoter-proximal (150 bp upstream of the TSS to 150 bp downstream of the TSS) regions for 9452 genes. The Spearman's correlation coefficients are shown in the plot. The gray diagonal lines represent a 1:1 fit. **(C, D)** Correlation plots between PRO-seq reads of biological replicates for the different time points after HS treatment in **(C)** gene body and **(D)** promoter-proximal regions for 9452 genes. The Spearman's correlation coefficients are shown in the plot. The gray diagonal lines represent a 1:1 fit.
