# Supplementary material for: Transcription factors GAF and HSF act at distinct regulatory steps to modulate stress-induced gene activation
Source: Genes Dev. 2016 Aug 1;30(15):1731–46. doi: 10.1101/gad.284430.116 (PMC5002978; doi:10.1101/gad.284430.116)
Supplement: Supplemental Material [file supp_gad.284430.116_Supplemental_FigureS8.pdf]

Rank	Motif's sequence logo	*MEME E*-value
1	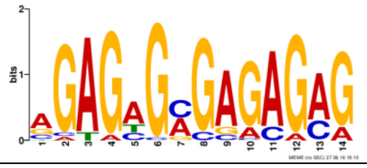	1.6×10^{-90}
2	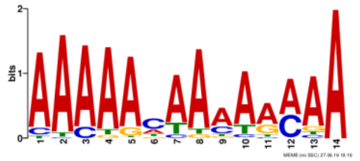	6.0×10^{-43}
3	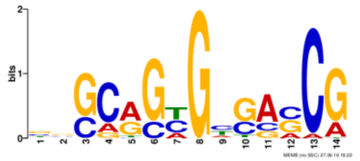	2.7×10^{-30}
4	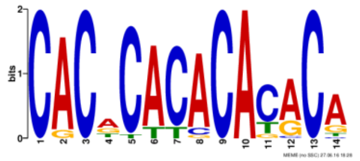	8.5×10^{-20}
5	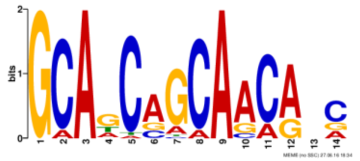	1.6×10^{-12}
6	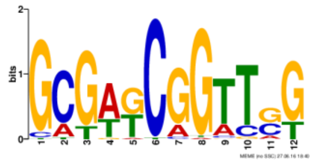	1.9×10^{-11}
7	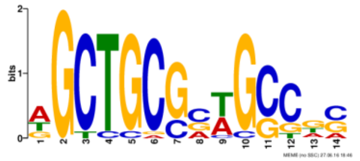	1.2×10^{-5}
8	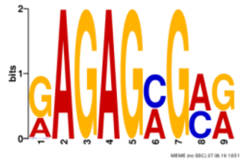	6.3×10^{-4}

Figure S8: De novo motif analysis of the promoter region of HS activated genes. Sequence logos and respective *E*-values generated by *MEME* (Bailey and Elkan 1994) of the motifs that were found enriched in the promoter region (-300 to +50 bp of the TSS) of HS activated genes. Motifs were ranked by *E*-value. The HSE, which varies in the arrangement of its critical 5 bp units, was not identified de novo by *MEME* in the promoter region of HS activated genes; however, individual matches to the HSE's position weight matrix were identified by *FIMO* (Grant et al. 2011) and were significantly enriched in the promoter region of HS activated genes relative to the repressed and unchanged classes.
