# Supplementary material for: Transcription factors GAF and HSF act at distinct regulatory steps to modulate stress-induced gene activation
Source: Genes Dev. 2016 Aug 1;30(15):1731–46. doi: 10.1101/gad.284430.116 (PMC5002978; doi:10.1101/gad.284430.116)
Supplement: Supplemental Material [file supp_gad.284430.116_Supplemental_FigureS12.pdf]

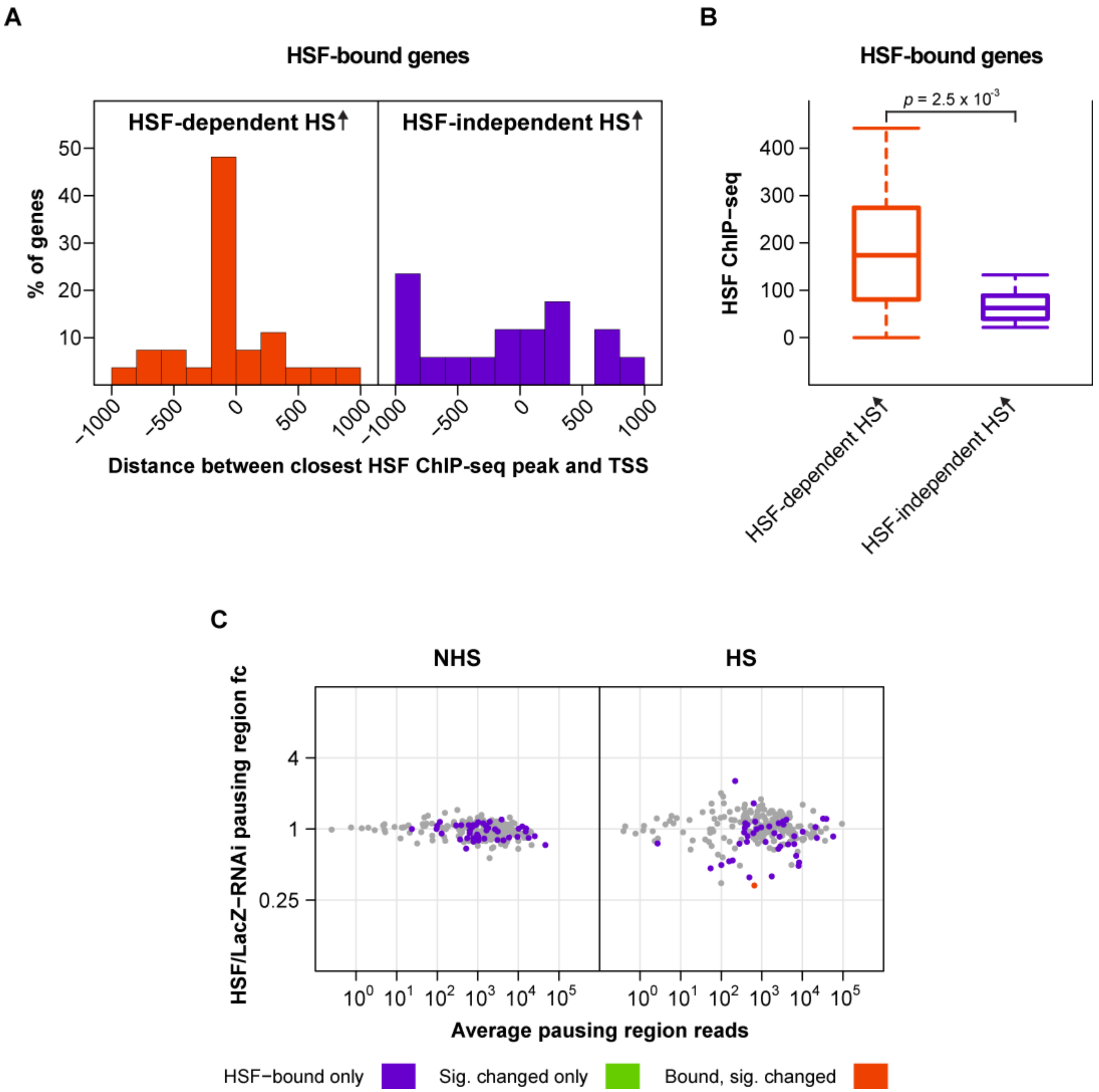

<u>Figure S12:</u> **Higher HSF binding levels and positioning upstream and proximal to the TSS are important for the induction of HSF's target genes. (A)** Histogram with the distribution of distances between the closest HSF ChIP-seq peak and the TSS of HSF-bound genes with HSF-dependent (left panel, n=27) or independent (right panel, n=17) HS activation. The distances are plotted in 200 bp bins from ±1000 bp to the TSS. **(B)** Box-plot showing the distribution of HSF ChIP-seq binding intensities for the two classes of genes described in A. Mann-Whitney *U* test p-value = 2.5×10^{-3}. **(C)** DESeq2 analysis to determine the effect of HSF-RNAi treatment on the PRO-seq pausing region reads before (NHS) and after 20min HS (HS). DESeq2 was used to identify significantly changed genes between HSF-RNAi and LacZ-RNAi cells and the results are displayed as MA plots. Significantly changed genes were defined using an FDR of 0.001. HSF−bound genes are labeled in purple, significantly changed genes (according to DESeq2) are labeled in green and genes that are both HSF-bound and significantly changed are labeled in orange.
