# Supplementary material for: Transcription factors GAF and HSF act at distinct regulatory steps to modulate stress-induced gene activation
Source: Genes Dev. 2016 Aug 1;30(15):1731–46. doi: 10.1101/gad.284430.116 (PMC5002978; doi:10.1101/gad.284430.116)
Supplement: Supplemental Material [file supp_gad.284430.116_Supplemental_FigureS6.pdf]

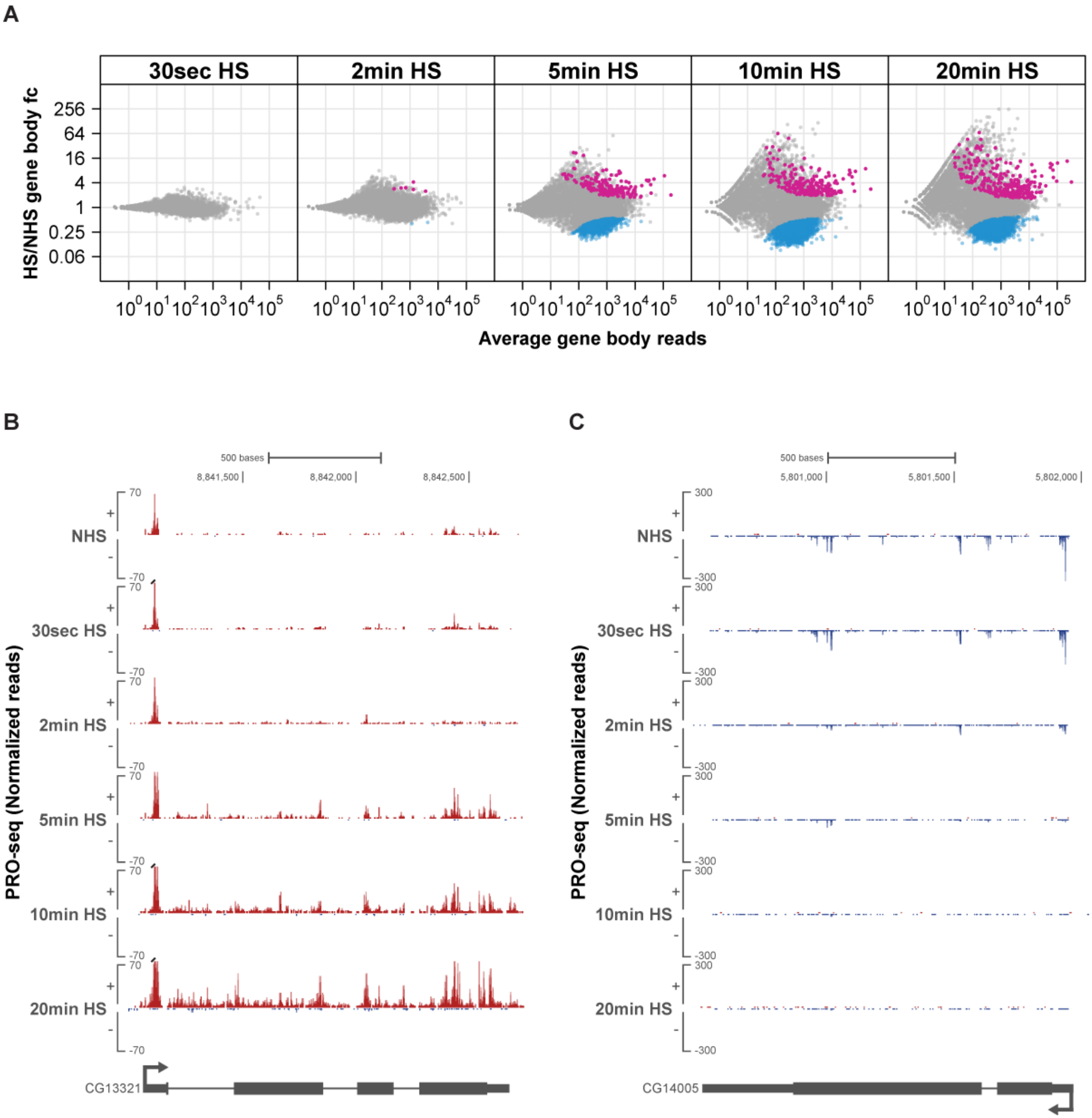

<u>Figure S6:</u> Substantial genome-wide response to HS occurs as early as 5 minutes post-HS. (A) DESeq2 analysis of PRO-seq gene body reads between HS-treated and NHS cells displayed as MA plots for the different time points after HS treatment. Significantly changed genes were defined using an FDR of 0.001. Activated genes are labeled in magenta and repressed genes in blue. fc = fold-change. **(B, C)** Representative view of a HS activated **(B)** and repressed **(C)** gene in the UCSC genome browser (Kent et al. 2002). PRO-seq normalized reads for the different time points for the plus strand are shown in red and for the minus strand in blue. Gene annotations are shown at the bottom.
