# Supplementary material for: Transcription factors GAF and HSF act at distinct regulatory steps to modulate stress-induced gene activation
Source: Genes Dev. 2016 Aug 1;30(15):1731–46. doi: 10.1101/gad.284430.116 (PMC5002978; doi:10.1101/gad.284430.116)
Supplement: Supplemental Material [file supp_gad.284430.116_Supplemental_FigureS10.pdf]

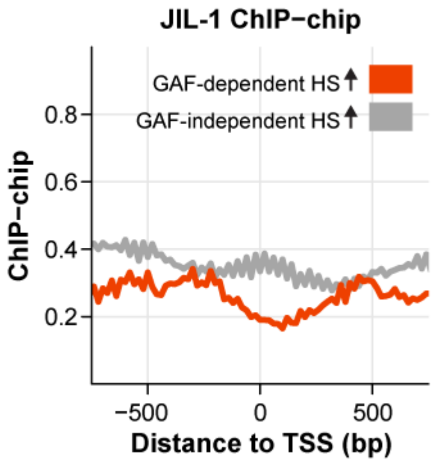

<u>**Figure S10:**</u> **Genes with GAF-dependent or GAF-independent HS activation have similar JIL-1 ChIP-chip profiles.** JIL-1 ChIP-chip signal between -750 to +750 bp to the TSS (in 20 bp bins) of genes with GAF-dependent (n=44) or GAF-independent (n=199) HS activation.
