# Supplementary material for: Transcription factors GAF and HSF act at distinct regulatory steps to modulate stress-induced gene activation
Source: Genes Dev. 2016 Aug 1;30(15):1731–46. doi: 10.1101/gad.284430.116 (PMC5002978; doi:10.1101/gad.284430.116)
Supplement: Supplemental Material [file supp_gad.284430.116_Supplemental_FigureS4.pdf]

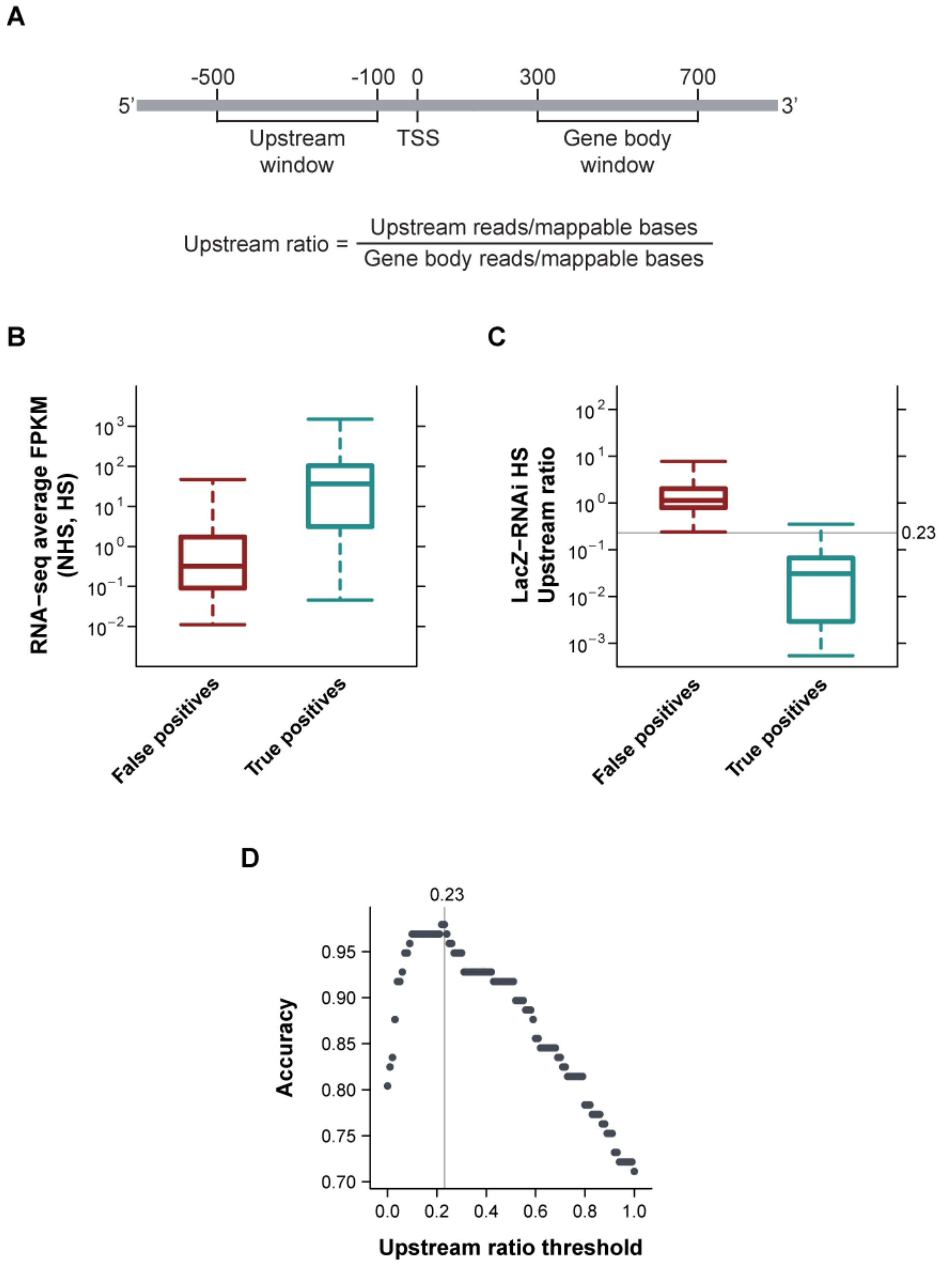

__Figure S4:__ **Validation of the upstream transcription filter implemented in our study. (A)** Diagram of the *upstream ratio* metric that was used to filter out false positive genes caused by run-through transcription from an upstream gene. **(B)** Box-plot of the average RNA−seq FPKM (NHS and HS) for the true (n=22) and false (n=78) positive subsets classified by visual inspection of 100 randomly selected activated genes. **(C)** Box-plot of the upstream ratio for the LacZ-RNAi HS condition for true and false positive genes. The 0.23 cutoff that was used to separate true from false positives is shown in the plot. **(D)** Accuracy metric ((true positives + true negatives)/total) of upstream ratio filter as a function of tested cutoffs. The cutoff with highest accuracy (0.23) is shown in the plot.
