# Supplementary material for: Transcription factors GAF and HSF act at distinct regulatory steps to modulate stress-induced gene activation
Source: Genes Dev. 2016 Aug 1;30(15):1731–46. doi: 10.1101/gad.284430.116 (PMC5002978; doi:10.1101/gad.284430.116)
Supplement: Supplemental Material [file supp_gad.284430.116_Supplemental_FigureS2.pdf]

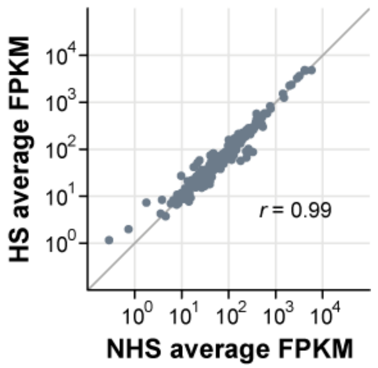

<u>**Figure S2:**</u> **mRNA levels of 335 genes used for normalization are not affected by HS.** Correlation plot between the RNA-seq FPKM for the NHS and HS conditions for the 335 HS-unaffected genes that were used to normalize our datasets. The FPKM values are the average of two biological replicates. The Pearson's correlation coefficient is shown in the plot. The gray line represents a 1:1 fit.
