# Supplementary material for: Transcription factors GAF and HSF act at distinct regulatory steps to modulate stress-induced gene activation
Source: Genes Dev. 2016 Aug 1;30(15):1731–46. doi: 10.1101/gad.284430.116 (PMC5002978; doi:10.1101/gad.284430.116)
Supplement: Supplemental Material [file supp_gad.284430.116_Supplemental_FigureS9.pdf]

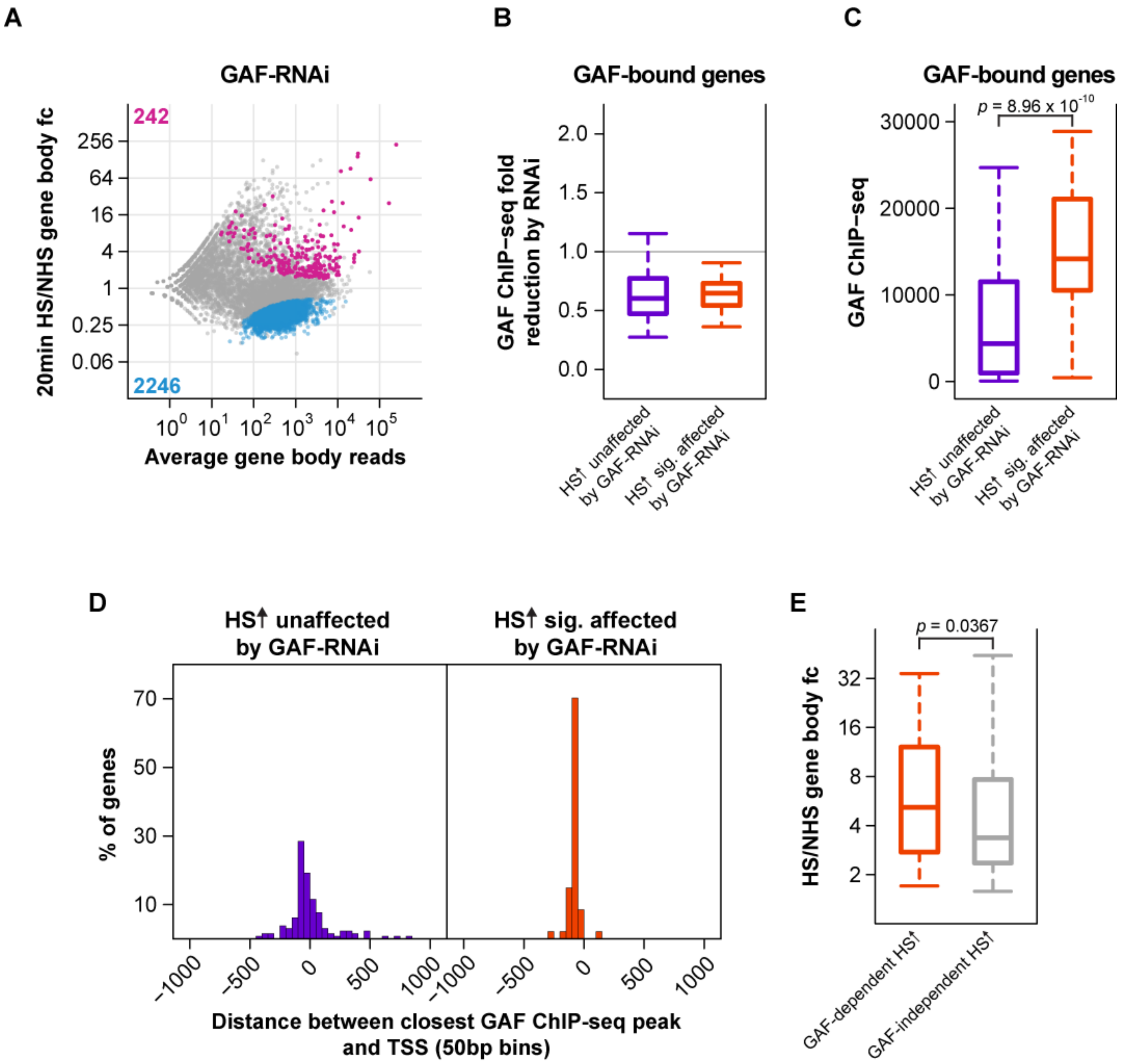

<u>Figure S9:</u> Higher binding levels and positioning immediately upstream of the core promoter are important for GAF's role in HS activation. (A) DESeq2 analysis of PRO-seq gene body reads of GAF-RNAi treated cells before (NHS) and after 20min HS displayed as an MA plot. Significantly changed genes were defined using an FDR of 0.001. Activated genes that passed our upstream transcription filter (see Materials and Methods) are labeled in magenta and repressed genes in blue. The number of genes in each class is shown in the plot. fc = fold-change. **(B)** Box-plot showing the fold-change of GAF ChIP-seq intensities after treatment with GAF-RNAi for GAF-bound genes whose HS induction is unaffected by GAF knockdown (purple, n=130) and GAF-bound genes whose HS induction is significantly affected by GAF knockdown (orange, n=47). **(C)** Box-plot showing the distribution of GAF ChIP-seq binding intensities for the two classes of genes described in B. Mann-Whitney *U* test p-value = 8.96×10^{-10}. **(D)** Histogram with the distribution of distances between the closest GAF ChIP-seq peak and the TSS of each gene in the two gene classes described in B. The distances are plotted in 50 bp bins from ±1000 bp to the TSS. **(E)** Box-plot showing the distribution of HS/NHS gene body fold-change for genes with GAF-dependent (n=44) or GAF-independent (n=199) HS activation. Mann-Whitney *U* test p-value = 0.0367.
