# Supplementary material for: Transcription factors GAF and HSF act at distinct regulatory steps to modulate stress-induced gene activation
Source: Genes Dev. 2016 Aug 1;30(15):1731–46. doi: 10.1101/gad.284430.116 (PMC5002978; doi:10.1101/gad.284430.116)
Supplement: Supplemental Material [file supp_gad.284430.116_Supplemental_FigureS13.pdf]

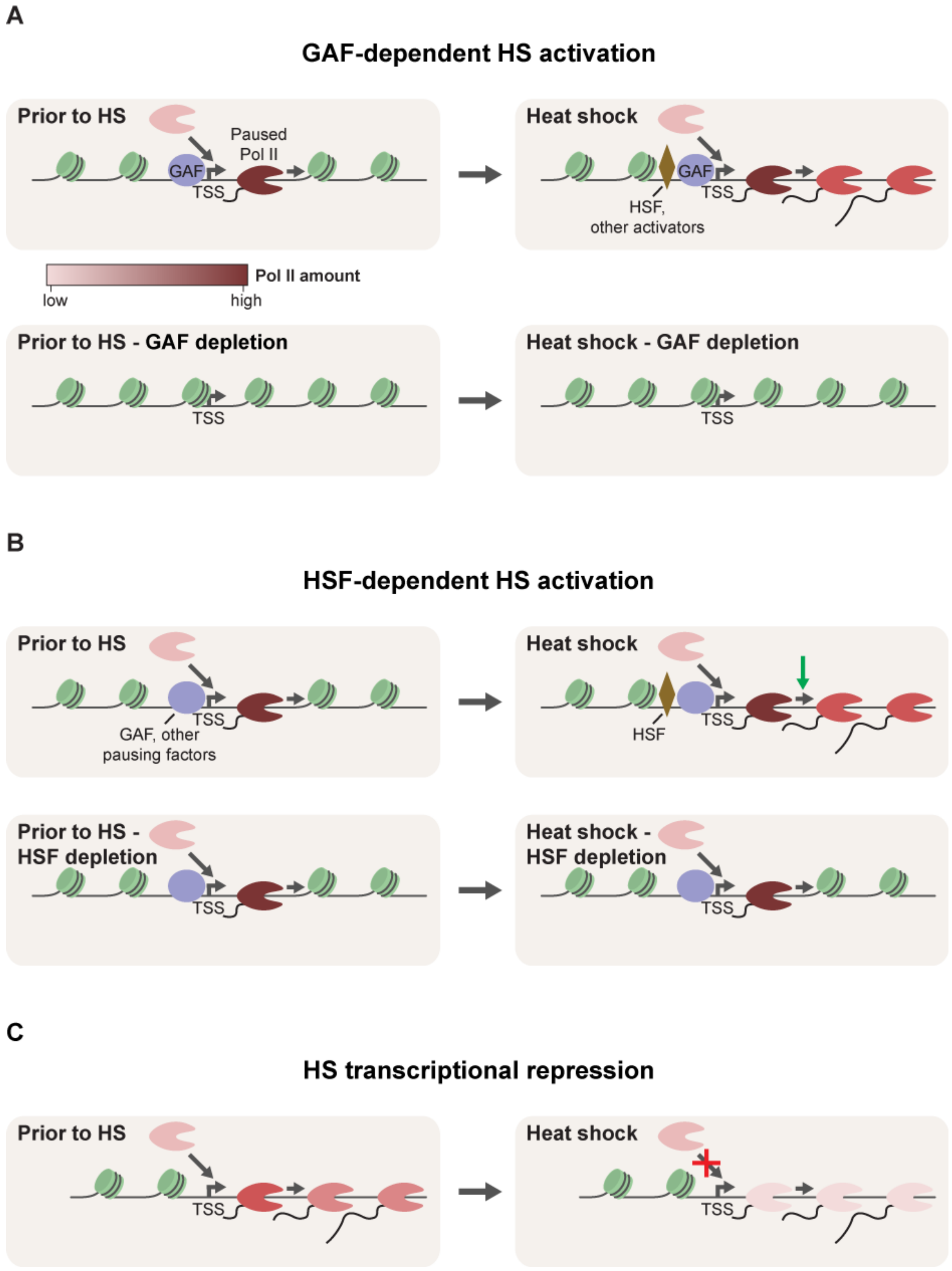

<u>Figure S13:</u> Summary of proposed mechanisms of HS transcriptional regulation. Model depicting the mechanisms of transcriptional regulation proposed in our study for **(A)** GAF-dependent HS activation, **(B)** HSF-dependent HS activation and **(C)** HS transcriptional repression. Red X represents a step that is being inhibited, and green arrow represents a step induced by HS. Nucleosomes are shown in green.
