# Supplementary material for: Transcription factors GAF and HSF act at distinct regulatory steps to modulate stress-induced gene activation
Source: Genes Dev. 2016 Aug 1;30(15):1731–46. doi: 10.1101/gad.284430.116 (PMC5002978; doi:10.1101/gad.284430.116)
Supplement: Supplemental Material [file supp_gad.284430.116_Supplemental_FigureS7.pdf]

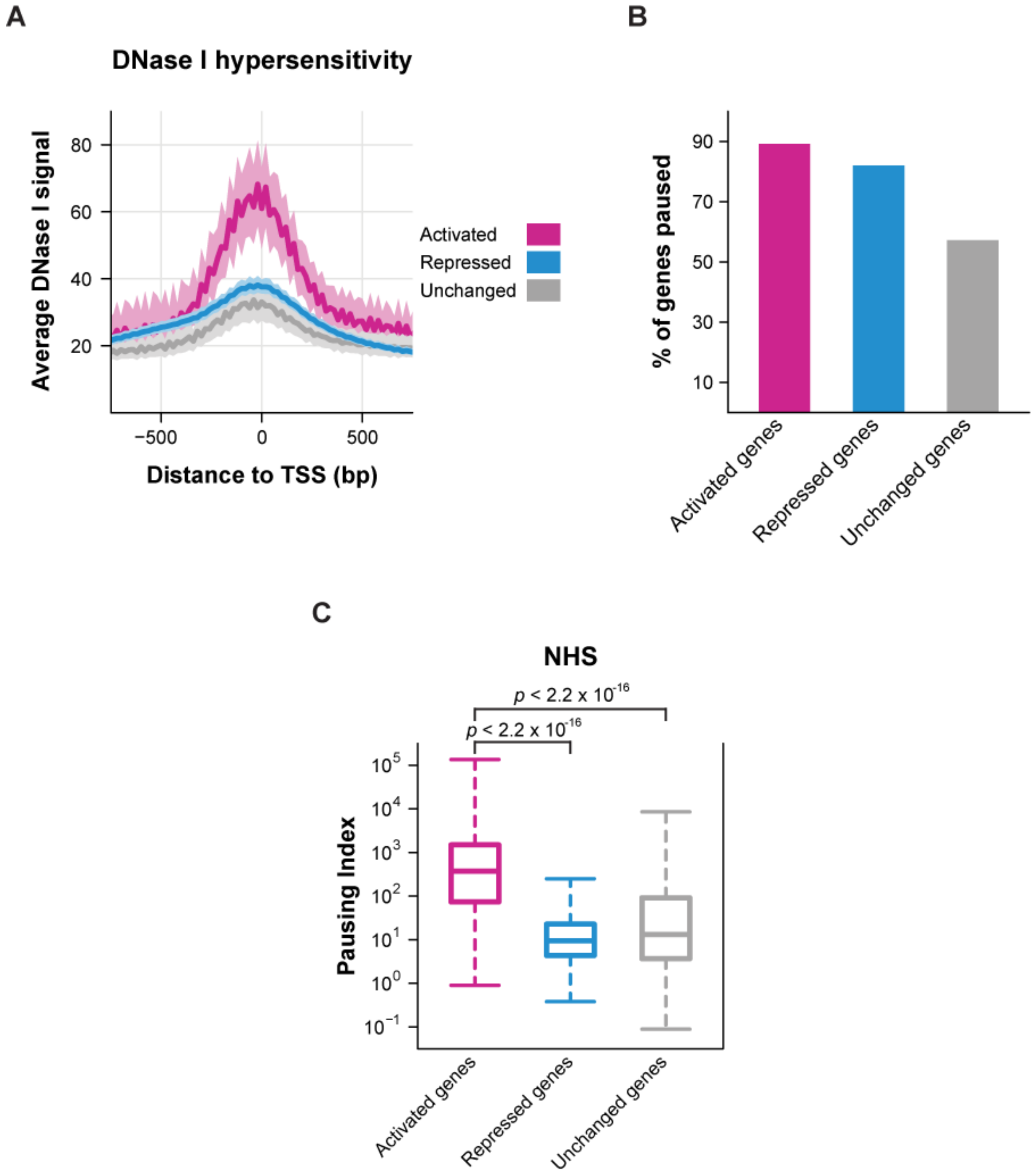

<u>Figure S7:</u> **Promoter region of HS activated genes is more accessible than repressed and unchanged classes prior to HS. (A)** DNase I hypersensitivity signal between -750 to +750 bp to the TSS (in 20 bp bins) of HS activated (n=249), repressed (n=2300), and unchanged (n=517) genes. The shaded area represents the 75% confidence interval. **(B)** Percentage of promoter-proximal paused genes in the HS activated, repressed, and unchanged classes. Paused genes were defined as the ones with significantly higher levels of read density in the promoter-proximal region relative to the gene body (Fisher's exact p-value <= 0.01) (Core et al. 2008). **(C)** Box-plot showing the LacZ-RNAi NHS pausing index distribution for HS activated, repressed, and unchanged genes. Mann-Whitney *U* test p-values are shown on the plot.
