# Supplementary material for: Transcription factors GAF and HSF act at distinct regulatory steps to modulate stress-induced gene activation
Source: Genes Dev. 2016 Aug 1;30(15):1731–46. doi: 10.1101/gad.284430.116 (PMC5002978; doi:10.1101/gad.284430.116)
Supplement: Supplemental Material [file supp_gad.284430.116_Supplemental_FigureS11.pdf]

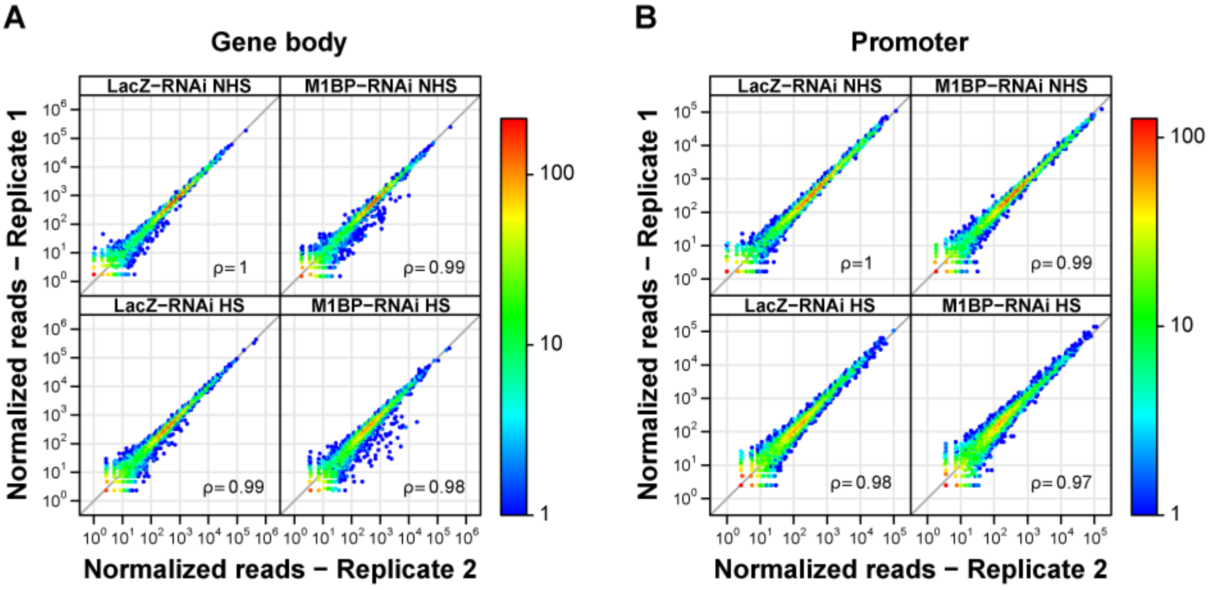

<u>Figure S11:</u> **Biological replicates of M1BP-RNAi and LacZ-RNAi control PRO-seq libraries were highly correlated for both promoter and gene body regions. (A, B)** Correlation plots between PRO-seq reads of biological replicates for the different RNAi treatments (LacZ and M1BP) in **(A)** gene body (200 bp downstream of the TSS to the polyadenylation site) and **(B)** promoter-proximal (150 bp upstream of the TSS to 150 bp downstream of the TSS) regions for 9452 genes. The Spearman's correlation coefficients are shown in the plot. The gray diagonal lines represent a 1:1 fit.
