# Supplementary material for: Transcription factors GAF and HSF act at distinct regulatory steps to modulate stress-induced gene activation
Source: Genes Dev. 2016 Aug 1;30(15):1731–46. doi: 10.1101/gad.284430.116 (PMC5002978; doi:10.1101/gad.284430.116)
Supplement: Supplemental Material [file supp_gad.284430.116_Supplemental_FigureS5.pdf]

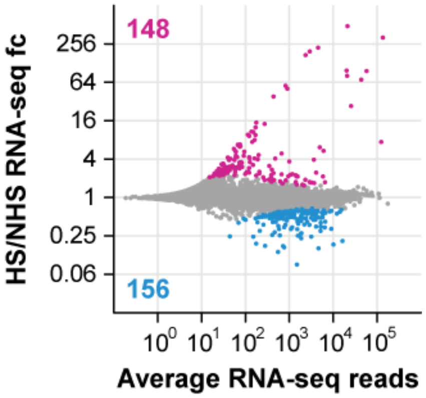

<u>Figure S5:</u> Measurement of steady-state mRNA levels by RNA-seq is unable to detect a genome-wide shutdown of transcription after HS. DESeq2 analysis of RNA-seq reads between 30min HS-treated and NHS cells displayed as an MA plot. Significantly changed genes were defined using an FDR of 0.001. Activated genes are labeled in magenta and repressed genes in blue. The number of genes in each class is shown in the plot. fc = fold-change.
