# Supplementary material for: Transcription factors GAF and HSF act at distinct regulatory steps to modulate stress-induced gene activation
Source: Genes Dev. 2016 Aug 1;30(15):1731–46. doi: 10.1101/gad.284430.116 (PMC5002978; doi:10.1101/gad.284430.116)
Supplement: Supplemental Material [file supp_gad.284430.116_Supplemental_TableS3.pdf]

Table S3: Sequencing and alignment of RNA-seq libraries.

For each replicate, the total number of reads sequenced, number of reads that passed filter, number of reads after clipping, number of reads that did not align to the ribosomal genes, and number of reads that aligned to the dm3 reference genome are shown in the table.

Library	Total reads	Passed filter	After clipping	Non-ribosomal	Mapped reads
**NHS_rep1**	46630399	33051180	27992112	24951251	21164958
**NHS_rep2**	29432765	20630727	17824656	17553278	15403098
**30minHS_rep1**	26881631	19246449	16839161	16345216	14286713
**30minHS_rep2**	29492622	20610831	18993871	18270231	15925348
