# Supplementary material for: Transcription factors GAF and HSF act at distinct regulatory steps to modulate stress-induced gene activation
Source: Genes Dev. 2016 Aug 1;30(15):1731–46. doi: 10.1101/gad.284430.116 (PMC5002978; doi:10.1101/gad.284430.116)
Supplement: Supplemental Material [file supp_gad.284430.116_Supplemental_FigureS3.pdf]

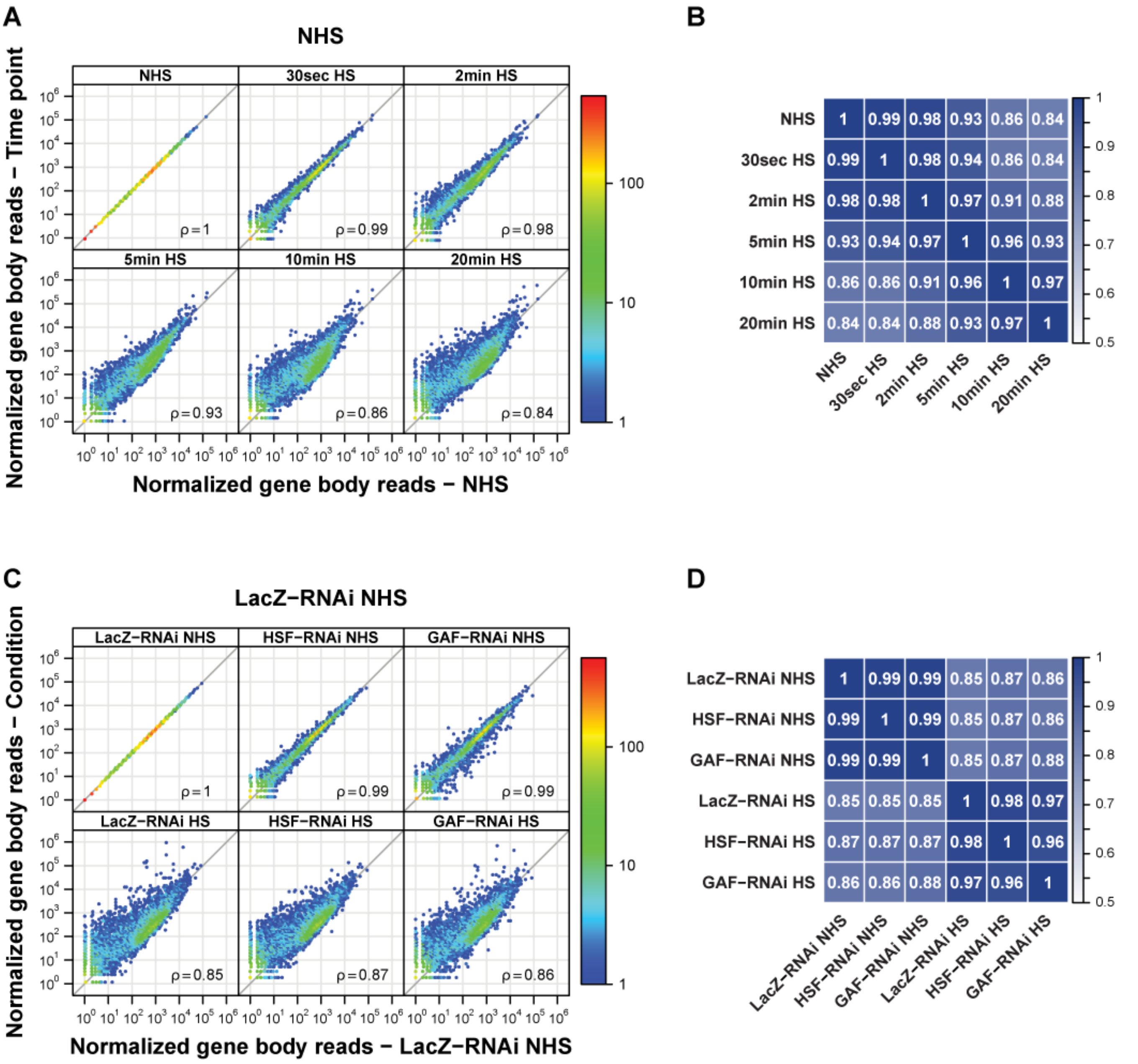

<u>Figure S3:</u> Validation of the PRO-seq normalization method used in our study. (A) Correlation plots between PRO-seq gene body reads of the NHS condition and all other time points after HS treatment for 9452 genes. The Spearman's correlation coefficients are shown in the plot. The gray lines represent a 1:1 fit. **(B)** Correlation matrix showing the Spearman's correlation coefficients for all combinations of time points. The correlation was calculated as in A for each individual sample. **(C)** Correlation plots between PRO-seq gene body reads of the LacZ-RNAi NHS control and all other treatments and conditions for 9452 genes. The Spearman's correlation coefficients are shown in the plot. The gray lines represent a 1:1 fit. **(D)** Correlation matrix showing the Spearman's correlation coefficients for all combinations of RNAi treatments (LacZ, HSF and GAF) and conditions (NHS and HS). The correlation was calculated as in C for each individual sample.
