# Supplementary material for: Transcription factors GAF and HSF act at distinct regulatory steps to modulate stress-induced gene activation
Source: Genes Dev. 2016 Aug 1;30(15):1731–46. doi: 10.1101/gad.284430.116 (PMC5002978; doi:10.1101/gad.284430.116)
Supplement: Supplemental Material [file supp_gad.284430.116_Supplemental_TableS1.pdf]

Table S1: Sequencing and alignment of PRO-seq libraries.

For each replicate, the total number of reads sequenced, number of reads that passed filter, number of reads after clipping, number of reads that did not align to the ribosomal genes, and number of reads that aligned uniquely to the dm3 reference genome are shown in the table.

Library	Total reads	Passed filter	After clipping	Non-ribosomal	Mapped reads
**LacZ_RNAi_NHS_rep1**	22002207	20498005	18857033	14282348	9443330
**LacZ_RNAi_NHS_rep2**	45852143	42614140	37948992	29125104	19052661
**LacZ_RNAi_20minHS_rep1**	38149191	35411265	32429537	26063433	12372995
**LacZ_RNAi_20minHS_rep2**	48224407	44752452	39159654	30083167	13759500
**GAF_RNAi_NHS_rep1**	30007019	27839237	25783567	18298791	11898316
**GAF_RNAi_NHS_rep2**	29223336	27156681	24128417	18720462	12469522
**GAF_RNAi_20minHS_rep1**	33083184	30743186	27869887	19884430	9288339
**GAF_RNAi_20minHS_rep2**	26900768	24906543	20804594	15242537	6899682
**HSF_RNAi_NHS_rep1**	31620891	29340277	26783955	19754947	12848598
**HSF_RNAi_NHS_rep2**	20661822	19223633	16847824	13244247	8850116
**HSF_RNAi_20minHS_rep1**	20303622	18904152	17287939	11342865	5292065
**HSF_RNAi_20minHS_rep2**	44198627	40832316	33567347	22388032	10971965
**M1BP_RNAi_NHS_rep1**	52108819	52108819	41939060	37382793	22434724
**M1BP_RNAi_NHS_rep2**	58684062	58684062	46843088	42896942	21490326
**M1BP_RNAi_20minHS_rep1**	41875931	41875931	30858958	28126011	13899422
**M1BP_RNAi_20minHS_rep2**	49215679	49215679	31324637	28638156	9749371
**LacZ_RNAi_NHS_rep1 (from M1BP-RNAi)**	46888842	46888842	38142007	32376920	20653749
**LacZ_RNAi_NHS_rep2 (from M1BP-RNAi)**	78247125	78247125	66730707	56231838	33911637
**LacZ_RNAi_20minHS_rep1 (from M1BP-RNAi)**	44850654	44850654	34314687	28456669	13326525
**LacZ_RNAi_20minHS_rep2 (from M1BP-RNAi)**	39884844	39884844	31949119	26644972	11580641
**NHS_rep1**	33326090	27513854	24770885	23263961	12081666
**NHS_rep2**	40386025	34851525	33127425	31427515	17083774
**30secHS_rep1**	32103863	26577493	24398076	23568732	13946293
**30secHS_rep2**	43868016	37825590	35476506	34014449	20066701
**2minHS_rep1**	29483939	24279233	21513613	20261645	12280319
**2minHS_rep2**	58929589	52883653	49114894	45522711	25046895
**5minHS_rep1**	26685698	22026373	20040132	18839288	10202616
**5minHS_rep2**	49420574	42648406	39527809	34244700	18258123
**10minHS_rep1**	31518051	26025041	23770378	22787845	10255055
**10minHS_rep2**	36364173	32508148	30318974	28531182	13973044
**20minHS_rep1**	53294616	47669950	44625103	42605985	19333234
**20minHS_rep2**	28918287	25102563	23838584	23118980	9322488
